# Combined Supplementation with Iron, Manganese and Zinc Sulfates Improves Growth and Modulates Leaf Gene Expression in Salt-Stressed *Rhizophora stylosa* Seedlings

**DOI:** 10.3390/biology15211933

**Published:** 2026-10-24

**Authors:** Jin Li, Qiaoxuan Li, Hongcheng Li, Xianmin Wang, Tingting Duan

**Affiliations:** 1 College of Chemistry and Environment, Guangdong Ocean University, Zhanjiang 524088, China; gdoujinli@gdou.edu.cn (J.L.);; 2 College of Coastal Agricultural Sciences, Guangdong Ocean University, Zhanjiang 524088, China

**Keywords:** mangrove seedlings, micronutrients, salinity, root architecture, oxidative injury, leaf transcriptome

## Abstract

*Rhizophora stylosa* seedlings were grown at three initial NaCl levels (0, 100 and 400 mM), with individual or combined iron, manganese and zinc sulfate supplementation at the two saline levels. Eleven treatments comprised three independent pots each. Growth, root architecture, photosynthesis, tissue elements and biochemical responses were assessed after 90 days. Relative to SCK, the high-salt control receiving basal nutrients without supplemental micronutrients, combined Fe + Mn + Zn (SFeMnZn) increased the dry mass by 29.8%, net photosynthetic rate by 67.0% and total root length by 133.1% (all adjusted *p* < 0.01). Maximum photosystem II photochemical efficiency increased from 0.659 to 0.795 (adjusted *p* < 0.001). Zn produced the strongest overall growth response among individual supplements, although their dry-mass differences were not significant. Combined supplementation increased tissue micronutrient concentrations, reduced oxidative injury and altered antioxidant and osmolyte responses. Leaf RNA sequencing of SFeMnZn versus SCK identified 1024 differentially expressed transcript entries associated with photosynthetic, redox, carbon-metabolic and cation-exchange processes. The findings support evaluation of the tested sulfate supplementation regime for controlled nursery cultivation and identify molecular candidates for further investigation.

## Simple Summary

Mangrove seedlings must be established in saline environments, where nutrient supply can influence growth. We studied *Rhizophora stylosa* seedlings at three initial NaCl levels (0, 100 and 400 mM), with iron, manganese and zinc supplied separately or together at the two saline levels. After 90 days, the combined Fe + Mn + Zn sulfate treatment produced larger seedlings, more extensive root systems, better photosynthetic performance and less oxidative injury than the high-salt control receiving basal nutrients alone. Zinc produced the strongest overall growth response among the individual supplements. Leaf and root Fe, Mn and Zn concentrations increased. Leaf gene-expression changes involved photosynthetic processes, redox metabolism, carbon metabolism and ion transport, providing candidate explanations for the physiological responses. These results support further development of the tested supplementation regime for controlled nursery cultivation and pre-transplant seedling conditioning.

## 1. Introduction

Rhizophora stylosa is a mangrove tree adapted to saline intertidal habitats. Understanding how mineral nutrition supports its seedling growth is relevant to both mangrove physiology and the production of planting material for restoration. Mangroves experience substantial variation in salinity [1], and moderate salinity can promote R. stylosa growth relative to fresh water, with the response depending on temperature [2]. These features make R. stylosa an ecologically relevant system for examining how nutrient supply influences growth and physiological performance under salt exposure.

Salt responses involve ion regulation, water relations, photosynthetic performance and cellular redox metabolism. In *Bruguiera parviflora*, salinity affects growth, ion accumulation, photosynthesis and leaf anatomy [3]. Experiments in *Kandelia obovata* connect improved performance under high salinity with K^+^/Na^+^ balance and transport-related gene expression [4], as well as antioxidant defense [5]. These findings support an integrated assessment of tissue elemental status, root development, photosynthesis and oxidative injury rather than reliance on a single stress marker.

Fe, Mn and Zn were selected because they support complementary functions that are relevant to these responses. Fe participates in electron-transfer and redox systems, while its uptake and intracellular allocation are tightly regulated [6]. Zn supports protein function and metabolic regulation and can influence plant stress responses [7]. Mn is required for the oxygen-evolving complex of photosystem II and for Mn-dependent enzymes [8]. Their distinct functions provide a rationale for testing both individual supplements and a combined supply; shared demands for uptake and cellular allocation also make the response to a mixture an empirical question. Supplemental Mn improves ion homeostasis and antioxidant responses in salt-treated rice [9], whereas micronutrient responses in mangroves can be dose-dependent [10]. Foliar Zn also improves water relations under salt treatment in pak choi, but that response depends on dose and application conditions [11]. The present study tests a supplementation response, rather than presuming that *R. stylosa* at the collection site was deficient in these three elements.

Transcriptome analysis can reveal which biological processes accompany a nutrient-treatment response. Joint physiological and transcriptomic work in *Sonneratia caseolaris* has identified salinity-associated antioxidant responses [12]. Here, leaf sequencing compares supplemented and unsupplemented seedlings within the same initial high-salt treatment. This contrast focuses on the response to the combined nutrient regime and complements organ-level biochemical measurements with candidate molecular processes.

We compared individual and combined Fe, Mn and Zn sulfate supplementation at initial NaCl levels of 100 and 400 mM, with a 0 mM reference. The local environmental context includes tidal-water salinities of 2–30 PSU at Gaoqiao and 15–30 PSU at Leizhou in the Zhanjiang Mangrove National Nature Reserve [13]. Published cultivation studies have also used 100 and 400 mM NaCl in *Bruguiera parviflora* and 400 mM NaCl in *Avicennia marina* [3,14]. These studies and our preliminary observations informed the selection of contrasting salt treatments. We asked which supplementation regimes improve seedling growth, how leaf and root responses differ, and which leaf transcriptional changes accompany the combined treatment. Root architecture, tissue elemental concentrations and physiological measurements were integrated to assess the response and its relevance to controlled nursery cultivation.

## 2. Materials and Methods

### 2.1. Plant Material and Cultivation

*Rhizophora stylosa* propagules were collected by the authors in June 2025 on Jinniu Island, Mazhang District, Zhanjiang, Guangdong, China (approximately 21.06° N, 110.25° E). The collected propagules were raised indoors, and seedlings of uniform size were selected for the experiment. Cultivation extended from June to December 2025, including seedling establishment and acclimation before the 90-day formal treatment period. No collection permit was required for this collection. Propagules were soaked in tap water for 3 days and planted in plastic pots measuring 60 × 20 × 25 cm, with drainage holes. Five seedlings were planted at equal spacing in a straight row in each pot. The substrate consisted entirely of river sand. River sand was treated with potassium permanganate and rinsed three times before use. Seedlings were acclimated with quarter-strength Hoagland nutrient solution without added NaCl until they reached the three-leaf stage and showed stable growth.

Greenhouse conditions were 30/24 °C day/night temperature and 75 ± 5% relative humidity, with natural light supplemented to a 14 h light/10 h dark photoperiod and an irradiance of 800–1000 µmol·m^−2^·s^−1^. These culture conditions were shared across treatments.

The plant material was identified as *Rhizophora stylosa* by Tingting Duan, a botanist and the corresponding author. A voucher specimen has been deposited in the Plant Taxonomy Laboratory, Guangdong Ocean University, under accession DuanT.TA153.

### 2.2. Treatments and Experimental Units

Eleven treatments were established at three initial NaCl levels (Table 1). WC received no added NaCl. At each saline level, the treatments comprised a control receiving basal nutrition, three individual micronutrient supplements and a combined Fe + Mn + Zn supplement. All treatments received the same quarter-strength Hoagland basal nutrient solution. CK denotes basal nutrition without additional micronutrient supplementation. L and S identify the initial 100 and 400 mM NaCl treatments; Lmix and Smix in the figures are abbreviations for LFeMnZn and SFeMnZn. The design compares supplementation strategies under saline cultivation; WC provides a no-added-NaCl reference.

Each treatment comprised three independent pots containing five seedlings each, giving 15 seedlings per treatment and 165 seedlings across 33 pots. The pot was the biological replicate and the unit used for statistical analysis. Plants within a pot were not treated as independent replicates. Treatment comparisons were evaluated within the resulting 11-group design.

Before the cultivation experiment, several field measurements in the Jinniu Island mangrove waters indicated salinities of approximately 3–25‰. The 100 and 400 mM NaCl treatments were selected by considering this local field range, published regional salinity and mangrove cultivation studies [3,13,14], and seedling growth in preliminary cultivation. These treatment concentrations correspond to approximately 5.84 and 23.38 g·L^−1^ NaCl, respectively, providing lower and higher initial salt exposures within the broad salt-content range encountered locally.

Supplement concentrations were selected using preliminary cultivation observations in which seedlings maintained normal growth, together with published plant studies of foliar ferrous sulfate and manganese sulfate supplementation [15,16]. Supplemental solutions contained 150 mg·L^−1^ FeSO_4_·7H_2_O, 312.5 mg·L^−1^ MnSO_4_·H_2_O and/or 138.9 mg·L^−1^ ZnSO_4_·H_2_O, corresponding to approximately 30 mg Fe·L^−1^, 100 mg Mn·L^−1^ and 50 mg Zn·L^−1^.The combined solution contained all three salts at these concentrations. The three sulfate salts were obtained from Aladdin (Shanghai, China). Low- and high-salt treatments received preprepared 100 and 400 mM NaCl solutions, respectively, at the start of the 90-day formal treatment period. Twelve supplementation applications were made at seven-day intervals on days 7–84. Each application comprised a 100 mL root drench and a 100 mL foliar spray per pot (20 mL per seedling), wetting both leaf surfaces evenly.

During the 90-day treatment period, each pot received 1.0 L of quarter-strength Hoagland nutrient solution without additional NaCl every two days. Each pot was managed against its initial reference mass. Collected drainage was returned to the original pot. Basal nutrient solution and recovered distilled-water rinses were included before weighing, after which distilled water was added as needed to restore the reference mass.

### 2.3. Sampling and Growth Measurements

Samples were collected on day 90, alternating among treatments. Fully expanded leaves from the second or third leaf pair below the apex were selected. Three seedlings were randomly selected within each pot for pooled leaf and root biochemical and elemental samples; equal fresh masses were contributed by the selected seedlings. Pot-level samples were processed separately. Leaf RNA samples were collected at the same time as the physiological samples. Biochemical samples were frozen in liquid nitrogen and stored at −80 °C.

Plant height and basal stem circumference were averaged across the five seedlings in each pot and expressed in centimeters. Root images were acquired with an IN-GX03 root analysis system(COMECAUSE, Weifang, China) and analyzed using WinRHIZO 2022b(Regent Instruments Inc., Québec, QC, Canada). The recorded traits were total root length (mm), surface area (mm^2^), volume (mm^3^), root tip number and fork number. Total root length is the summed length of all detected root segments, including fine and lateral roots. Three randomly selected seedlings per pot were scanned, and their measurements were averaged to represent that pot. These three seedlings supplied the destructive tissue samples. The other two intact seedlings were used for whole-plant dry mass: their roots, stems and leaves were collected, heated at 105 °C for 30 min and dried at 75 °C to constant mass. Constant mass was defined as a relative change below 0.1% between successive weighings after further drying and cooling. The combined dry mass of the two seedlings was divided by two to obtain the mean single-plant dry mass for each pot. Treatment means and sample SDs were calculated from three independent pot means (n = 3).

### 2.4. Gas Exchange, Photosynthetic Pigments and Chlorophyll Fluorescence

Net photosynthetic rate (Pn), stomatal conductance (Gs), intercellular CO_2_ concentration (Ci) and transpiration rate (Tr) were measured with an LI-6400XT system (LI-COR Biosciences, Lincoln, NE, USA) [17] on clear days between 09:00 and 11:00 before harvest. Chamber conditions were 1200 µmol photons·m^−2^·s^−1^, 400 µmol CO_2_·mol^−1^ and 30 °C. One third of a fully expanded leaf per seedling was measured, and five seedling measurements were averaged per pot. Measurements were taken from the middle third of the third fully expanded leaf, using a consistent position within the leaf chamber.

After removal of the main veins, 0.100 g of fresh leaf tissue was cut into small pieces and extracted with 80% (*v*/*v*) acetone in darkness until decolorized. The extract was brought to 10.0 mL. Following centrifugation, absorbance was measured at 663.2, 646.8 and 470 nm against an 80% acetone blank. Concentrations were calculated as Ca = 12.25A663.2 − 2.79A646.8, Cb = 21.50A646.8 − 5.10A663.2 and Ccar = (1000A470 − 1.82Ca − 85.02Cb)/198 [18]. Total chlorophyll was calculated as Ca + Cb. Concentrations were converted to mg·g^−1^ fresh weight (FW) as C × V × D/(1000 m), where V is extract volume in mL, D is any applicable dilution factor and m is tissue fresh mass in g.

Maximum photosystem II (PSII) photochemical efficiency was measured for WC, LCK, LFeMnZn, SCK and SFeMnZn using an Imaging-PAM M-Series system (Heinz Walz GmbH, Effeltrich, Germany). Leaves were dark-adapted for 30 min to record Fo, followed by a saturating pulse of approximately 2700 µmol photons·m^−2^·s^−1^ for 0.8 s to record Fm. Fv/Fm was calculated as (Fm − Fo)/Fm [19]. The whole-leaf region of interest was used to calculate mean Fv/Fm after excluding the background. One leaf from each of three randomly selected seedlings per pot was measured, and the three leaf values were averaged to represent that pot. Three independent pots were measured per treatment. Representative images were selected from leaves with values close to their treatment means; inferential statistics used the pot-level values.

### 2.5. Tissue Elements, Osmolytes and Redox Measurements

Leaf and root samples were heated at 105 °C for 30 min and dried at 75 °C to a constant mass (<0.1% relative mass change). Ground samples (0.2000 g) were microwave-digested with 5.0 mL HNO_3_ and 1.0 mL 30% H_2_O_2_. Fe, Mn and Zn were measured by inductively coupled plasma mass spectrometry (ICP-MS)(7500cx, Agilent Technologies, Santa Clara, CA, USA) and expressed as mg·kg^−1^ dry weight (DW). NIST SRM 1573a (Tomato Leaves) served as the quality-control reference material [20].

Soluble sugars were determined by anthrone colorimetry [21]. Proline was extracted with 3% sulfosalicylic acid and determined by acid-ninhydrin colorimetry at 520 nm [22]. Reduced glutathione (GSH) was assayed with 5,5′-dithiobis (2-nitrobenzoic acid) (DTNB) at 412 nm after extraction with 5% sulfosalicylic acid, using DTNB thiol colorimetry [23]. Results were expressed as mg·g^−1^ FW for sugars, µg·g^−1^ FW for proline and µmol·g^−1^ FW for glutathione.

Superoxide dismutase (SOD), peroxidase (POD) and catalase (CAT) were extracted in cold phosphate buffer; the ascorbate peroxidase (APX) extraction buffer additionally contained ascorbate. SOD was measured by inhibition of nitroblue tetrazolium (NBT) photoreduction at 560 nm [24], POD by guaiacol oxidation at 470 nm [25], CAT by H_2_O_2_ disappearance at 240 nm [26], and APX by ascorbate oxidation at 290 nm [27]. Polyphenol oxidase (PPO) was measured by catechol oxidation at 420 nm [28]. Activities were expressed as U·g^−1^ FW. One SOD unit produced 50% inhibition of NBT photoreduction; one POD, CAT or APX unit represented formation or consumption of 1 µmol of the respective product or substrate per minute. For this study, one PPO unit was defined as an A420 increase of 0.01 min^−1^. Extraction and reaction conditions are provided in Supplementary Methods S1.

Malondialdehyde (MDA) was assayed using a modified thiobarbituric acid–trichloroacetic acid (TBA–TCA) assay for lipid peroxidation [29], with absorbance recorded at 450, 532 and 600 nm. H_2_O_2_ was measured by potassium iodide (KI) colorimetry at 390 nm [30], and superoxide production by hydroxylamine oxidation at 530 nm [31]. MDA was expressed as nmol·g^−1^ FW and H_2_O_2_ as µmol·g^−1^ FW, and superoxide production as nmol·g^−1^ FW·min^−1^. Relative electrolyte conductivity (REC) was determined by conductivity measurement and expressed as a percentage [29].

Leaf 3,3′-diaminobenzidine (DAB) and NBT staining provided qualitative observations alongside the biochemical assays using adapted histochemical procedures [32]. Roots were labeled with 10 µM CM-H_2_DCFDA for 20 min at 25 °C in darkness and imaged using a Leica M165 FC (Leica Microsystems, Heerbrugg, Switzerland) with the GFP plants filter set (excitation 450–490 nm; emission 500–550 nm). Acquisition conditions were held constant across treatments (150 ms exposure, gain 1.0×, gamma 1.0). Scale bars represent 200 µm, corresponding to 130 source-image pixels (approximately 1.538 µm·pixel^−1^). The probe-based approach follows the principle of root CM-H_2_DCFDA imaging [33], with the incubation and acquisition settings specified here. Fluorescence was treated as a qualitative indicator of oxidative activity.DAB, NBT and CM-H2DCFDA were obtained from Solarbio (Beijing, China). Detailed staining procedures are given in Supplementary Methods S1.

### 2.6. Leaf RNA Sequencing and Functional Analysis

Leaf RNA sequencing compared SCK and SFeMnZn under the initial 400 mM NaCl treatment. One pooled sample from three selected seedlings represented each pot; three pots per treatment underwent separate RNA extraction and library preparation. Report labels S1–S3 and W1–W3 correspond to SCK and SFeMnZn, respectively.

RNA concentration was determined fluorometrically using an instrument manufactured by Shanghai Jinxin Biotechnology Co., Ltd. (Shanghai, China), and RNA integrity was assessed using a Qsep400 analyzer (BiOptic Inc., New Taipei City, Taiwan). Following mRNA enrichment, cDNA synthesis, library preparation and quality control, DNA nanoball libraries were sequenced through the MGI transcriptome service (MGI Tech Co., Ltd., Shenzhen, China). Reads were filtered using fastp 0.23.2 [34]. The reported filters removed adapter-containing reads, read pairs with more than 10% ambiguous bases in either read, and pairs in which more than 50% of bases in either read had a quality score of 20 or lower.

Clean reads were assembled using Trinity v2.15.1 [35] and clustered with Corset 1.09 to generate unigenes. Functional annotation used Diamond v2.0.9 and the KEGG, Nr, SwissProt, TrEMBL, KOG, GO and Pfam databases. Bowtie2 2.4.5 and RSEM v1.3.1 were used for alignment and expression quantification [36]. Differential expression was evaluated from read counts using DESeq2 1.22.2 [37], with |log_2_ fold change| ≥ 1 and Benjamini–Hochberg-adjusted *p* < 0.05. Throughout this article, a positive fold change denotes higher expression in SFeMnZn than in SCK.

GO and KEGG overrepresentation analysis followed the sequencing service workflow using hypergeometric tests. Report-derived enrichment displays were retained, with cosmetic adjustments documented in the figure captions. Pearson sample correlations and principal component analysis were used to describe expression-profile relationships among the six leaf libraries (Figure S3). Candidate transcripts were screened in NR and Swiss-Prot annotations using four keyword groups: photosystem/chlorophyll a-b/protochlorophyllide; peroxidase/glutathione S-transferase/gamma-glutamylcyclotransferase/glucose-6-phosphate 1-dehydrogenase; proline transporter/sucrose-phosph/phosphoglucomutase; and cation/proton or cation/calcium exchanger. All 44 matching entries were retained, including opposing responses and one flagged annotation conflict. These functional groups were defined by annotation and relevance to the physiological measurements, not by expression clustering. Expression was displayed as row z-scores of log_2_(FPKM + 1) across six samples. Cluster identifiers were preserved; multiple entries need not represent independent gene loci.

The raw RNA-seq reads have been deposited in the NCBI Sequence Read Archive (SRA) under BioProject accession PRJNA1538750. The SRA run accessions are SRR41004780 (SCK1), SRR41004779 (SCK2), SRR41004778 (SCK3), SRR41004777 (SFeMnZn1), SRR41004776 (SFeMnZn2), and SRR41004775 (SFeMnZn3).

### 2.7. Statistical Analysis

The pot was the experimental unit (n = 3 independent pots per treatment). Means and sample standard deviations were calculated from the original pot-level values. Welch one-way ANOVA was followed by Games–Howell comparisons at α = 0.05: 55 pairs among 11 treatments per endpoint, except Fv/Fm, which comprised 10 pairs among five measured treatments. No additional Holm correction was applied. All 2540 pairwise decisions across 47 endpoints were checked against the compact letter displays. Letters are compared within one endpoint only: no common letter indicates adjusted *p* < 0.05, whereas a common letter indicates that a significant difference was not detected. Total chlorophyll is a derived endpoint. Figures display all observations and mean ± SD; any retained axis break omits an interval without observations or SD limits. Percentage changes were calculated from unrounded means. Transcriptome differential-expression inference was taken from the DESeq2 analysis and was not recalculated using physiological tests or FPKM values.

### 2.8. AI-Assisted Manuscript Preparation

During manuscript preparation, the authors used AI tools from OpenAI to assist with journal-specific formatting, reference organization, and English language polishing of the main text. These tools were not used to generate new experimental data during manuscript preparation. The authors reviewed and edited the output and take full responsibility for the entire content of the final manuscript.

## 3. Results

### 3.1. Growth and Root Architecture

**Figure 1:**
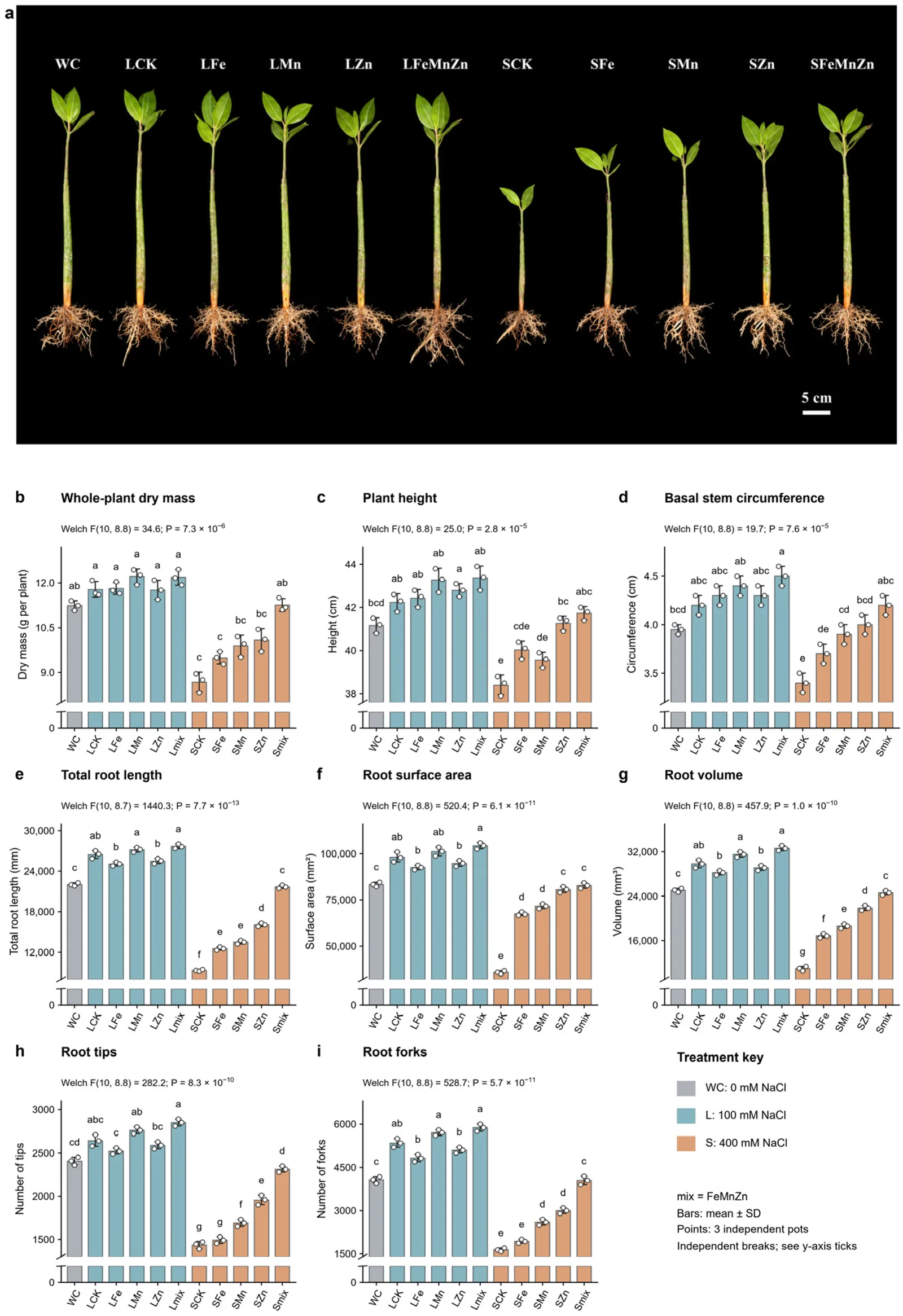
Growth and root architecture. (**a**) Representative seedlings; (**b**) whole-plant dry mass; (**c**) plant height; (**d**) basal stem circumference; (**e**) total root length; (**f**) root surface area; (**g**) root volume; (**h**) root tip number; (**i**) root fork number.Bars show means ± SD; points represent three independent pots. Welch ANOVA results are shown. Letters are compared only within each endpoint using Games–Howell tests (α = 0.05); groups with no shared letter differ significantly. WC, 0 mM NaCl; L, 100 mM; S, 400 mM; Lmix and Smix, LFeMnZn and SFeMnZn. Panel-specific y-axis breaks omit empty basal intervals; zero baselines, all observations and complete SD limits are retained. Residual bar lengths across breaks should not be used to infer ratios. The supplied photograph is reproduced at a uniform scale.

SFeMnZn increased the dry mass, height and stem circumference relative to SCK by 29.8%, 8.7% and 23.5%, respectively (all Games–Howell-adjusted *p* < 0.01; Figure 1). The total root length, surface area and volume increased by 133.1%, 131.1% and 125.2%; root tip and fork numbers increased by 61.2% and 147.3% (all adjusted *p* < 0.01). The high-salt control had lower values than WC for all eight growth endpoints. At 100 mM NaCl, none of the eight LFeMnZn–LCK growth comparisons reached significance; root volume had adjusted *p* = 0.0599. Among the individual high-salt supplements, SZn had the highest mean values across the eight growth endpoints. It exceeded SFe and SMn in total root length, surface area, volume and root tip number; dry mass did not differ significantly among the three single-element treatments.

### 3.2. Leaf and Root Micronutrient Concentrations

Combined supplementation increased all six tissue-element concentrations relative to SCK (Table 2; all adjusted *p* < 0.01). Leaf Fe, Mn and Zn increased by 98.4%, 243.4% and 226.7%, respectively; the corresponding root increases were 96.6%, 195.5% and 252.8%. The organ-resolved measurements demonstrate changes in tissue nutrient concentrations, which are reported separately from whole-plant biomass.

### 3.3. Gas Exchange, Pigments and PSII Efficiency

**Figure 2:**
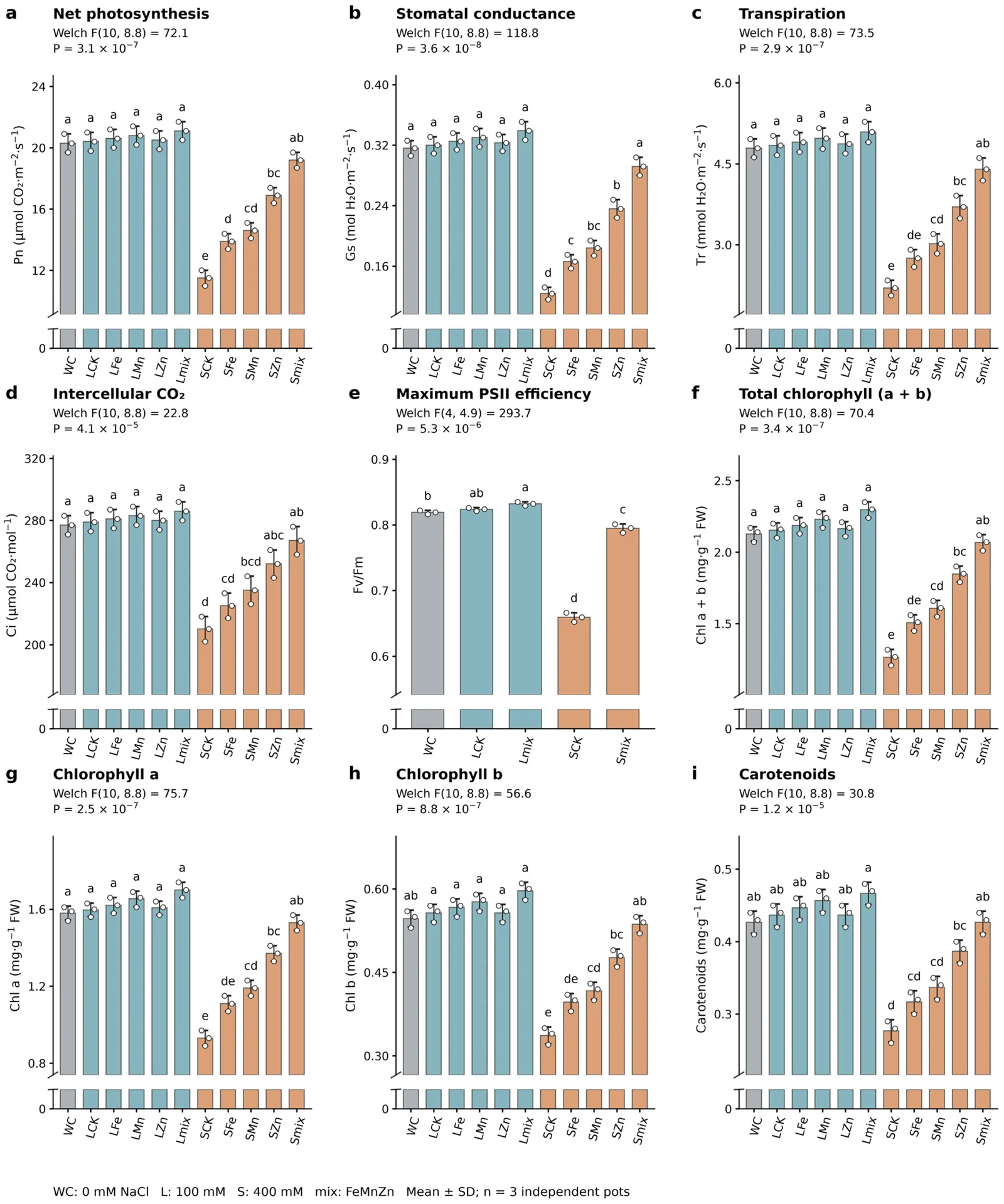
Gas exchange, pigments and PSII efficiency. (**a**) Net photosynthetic rate; (**b**) stomatal conductance; (**c**) transpiration rate; (**d**) intercellular CO_2_ concentration; (**e**) maximum PSII photochemical efficiency (Fv/Fm); (**f**) total chlorophyll; (**g**) chlorophyll a; (**h**) chlorophyll b; (**i**) carotenoids. Bars show means ± SD; points represent three independent pots. Welch ANOVA results are shown. Letters are compared only within each endpoint using Games–Howell tests (α = 0.05); groups with no shared letter differ significantly. WC, 0 mM NaCl; L, 100 mM; S, 400 mM; Lmix and Smix, LFeMnZn and SFeMnZn. Fv/Fm includes only five measured treatments; all other panels include 11 treatments. Total chlorophyll is derived from chlorophyll a and b. Marked breaks omit empty intervals.

**Figure 3:**
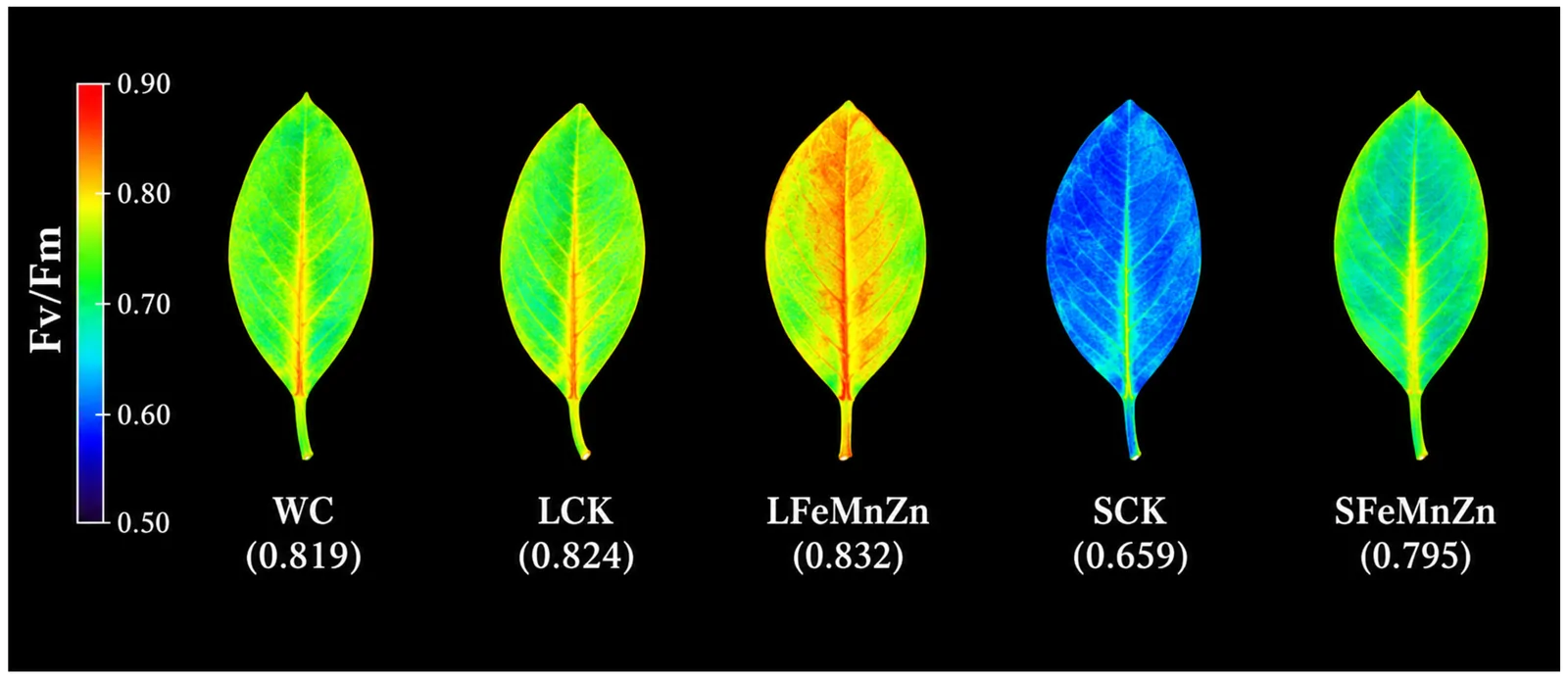
Representative Fv/Fm images for WC, LCK, LFeMnZn, SCK and SFeMnZn, matching the five treatments in the quantitative Fv/Fm panel. The original color scale and annotated values are retained. Images illustrate PSII maximum photochemical efficiency and do not constitute additional biological replicates; treatment significance is based on the three independent pot-level measurements.

Relative to SCK, SFeMnZn increased Pn by 67.0%, Gs by 135.5%, Ci by 27.1% and Tr by 100.0% (Figure 2; all adjusted *p* < 0.05). Chlorophyll a, chlorophyll b and carotenoids increased by 64.5%, 59.4% and 54.2%, respectively (all adjusted *p* < 0.01). Derived total chlorophyll increased by 63.2%. Fv/Fm increased from 0.659 to 0.795 (adjusted *p* < 0.001). The LFeMnZn–LCK comparison for Fv/Fm was not significant (adjusted *p* = 0.0935). Representative Fv/Fm images show lower PSII maximum photochemical efficiency in SCK and higher values in SFeMnZn (Figure 3), consistent with the pot-level measurements. The images indicate photochemical function, not chloroplast ultrastructure.

### 3.4. Osmolyte Concentrations

**Figure 4:**
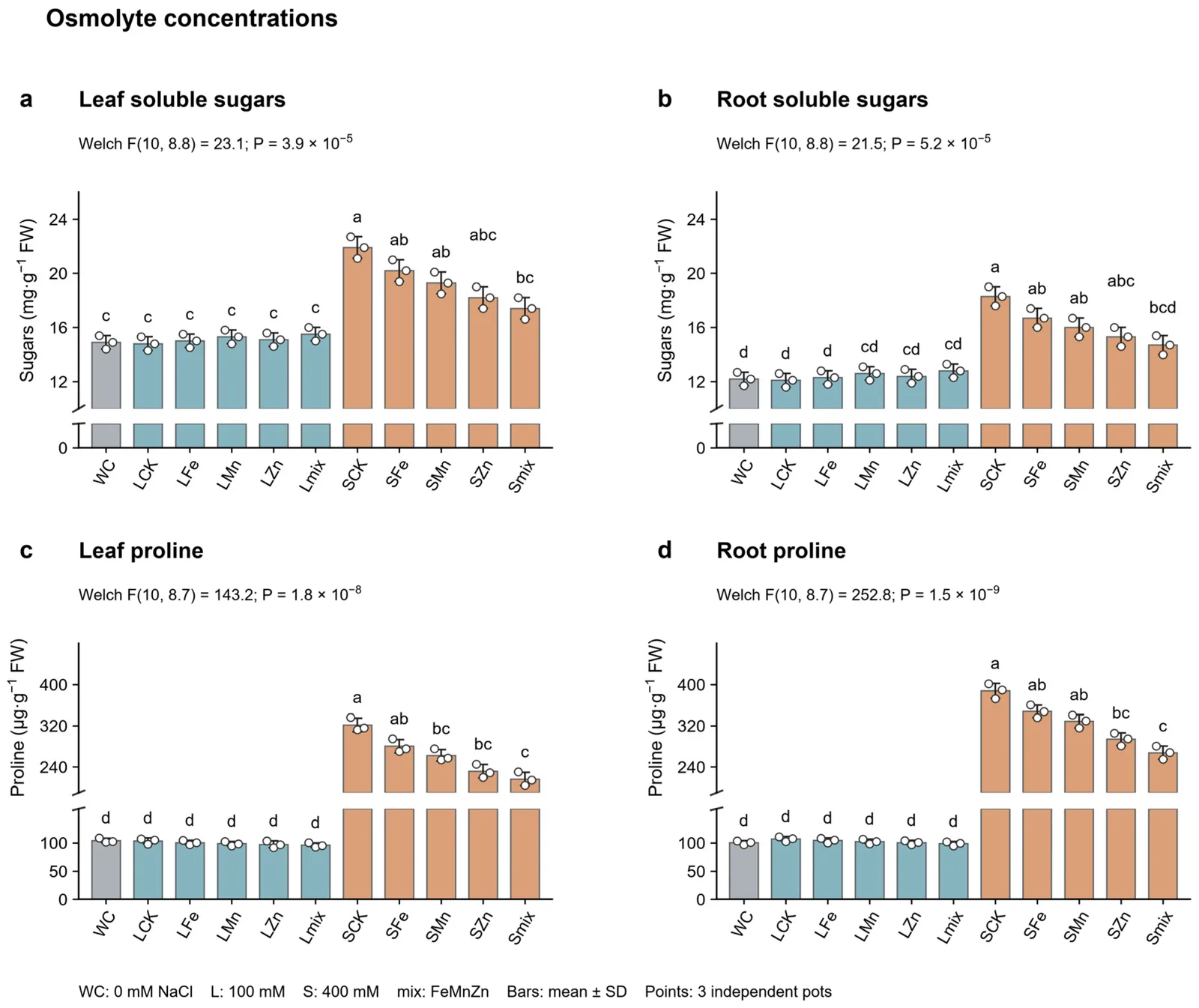
Leaf and root soluble sugars and proline. (**a**) Leaf soluble sugars; (**b**) root soluble sugars; (**c**) leaf proline; (**d**) root proline. Bars show means ± SD; points represent three independent pots. Welch ANOVA results are shown. Letters are compared only within each endpoint using Games–Howell tests (α = 0.05); groups with no shared letter differ significantly. WC, 0 mM NaCl; L, 100 mM; S, 400 mM; Lmix and Smix, LFeMnZn and SFeMnZn. Marked breaks omit intervals without observations or SD limits.

SFeMnZn lowered soluble sugars by 20.5% in leaves and 19.7% in roots relative to SCK (adjusted *p* = 0.0254 and 0.0349); proline decreased by 32.6% and 31.0% (adjusted *p* = 0.0065 and 0.0051; Figure 4). These changes occurred alongside improved growth and photosynthetic performance.

### 3.5. Oxidative Injury and Membrane Integrity

**Figure 5:**
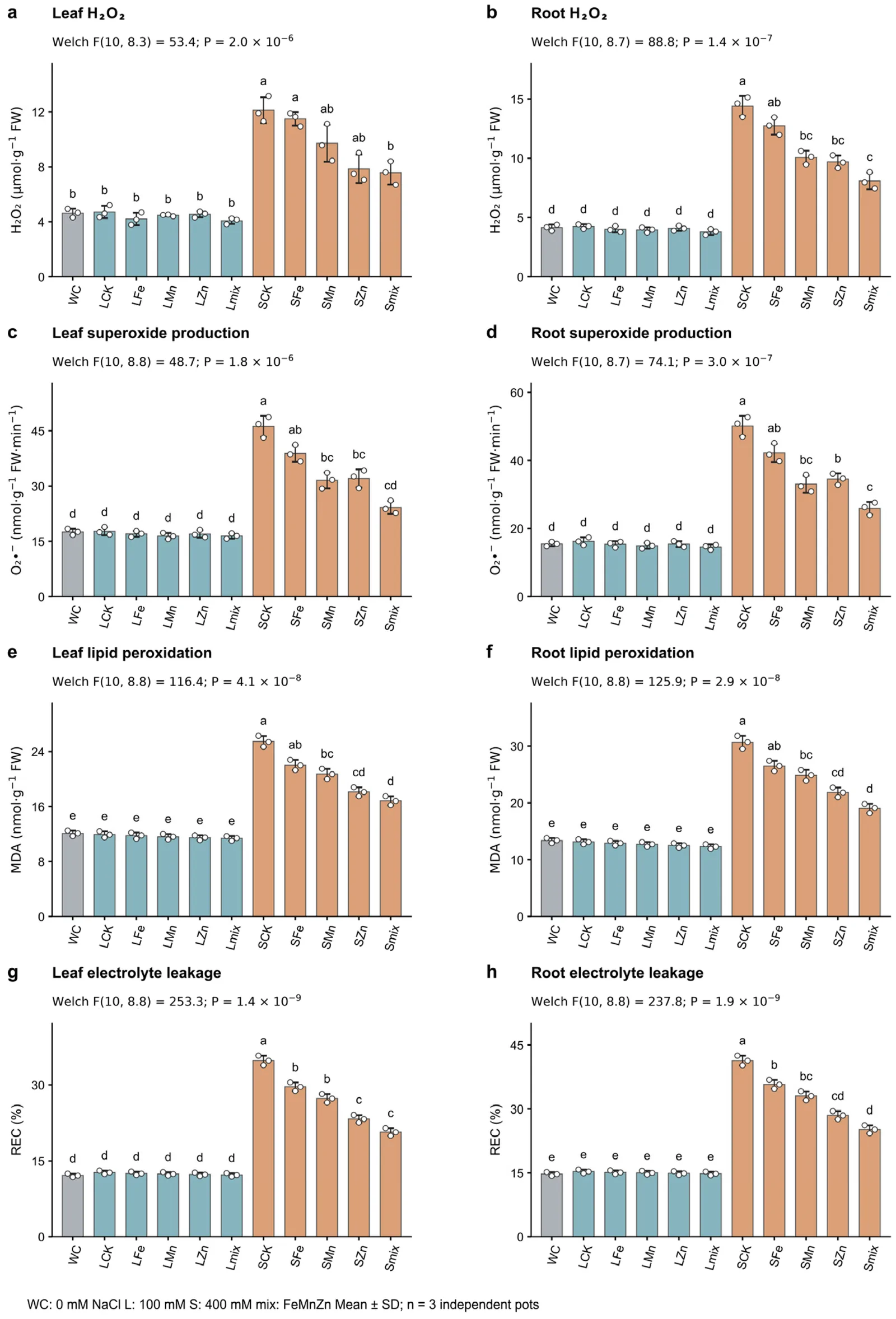
Leaf and root reactive oxygen species (ROS), lipid peroxidation and electrolyte leakage. (**a**) Leaf H_2_O_2_; (**b**) root H_2_O_2_; (**c**) leaf superoxide production; (**d**) root superoxide production; (**e**) leaf malondialdehyde (MDA); (**f**) root MDA; (**g**) leaf relative electrolyte conductivity (REC); (**h**) root REC. Bars show means ± SD; points represent three independent pots. Welch ANOVA results are shown. Letters are compared only within each endpoint using Games–Howell tests (α = 0.05); groups with no shared letter differ significantly. WC, 0 mM NaCl; L, 100 mM; S, 400 mM; Lmix and Smix, LFeMnZn and SFeMnZn. All eight panels have continuous zero-based axes.

**Figure 6:**
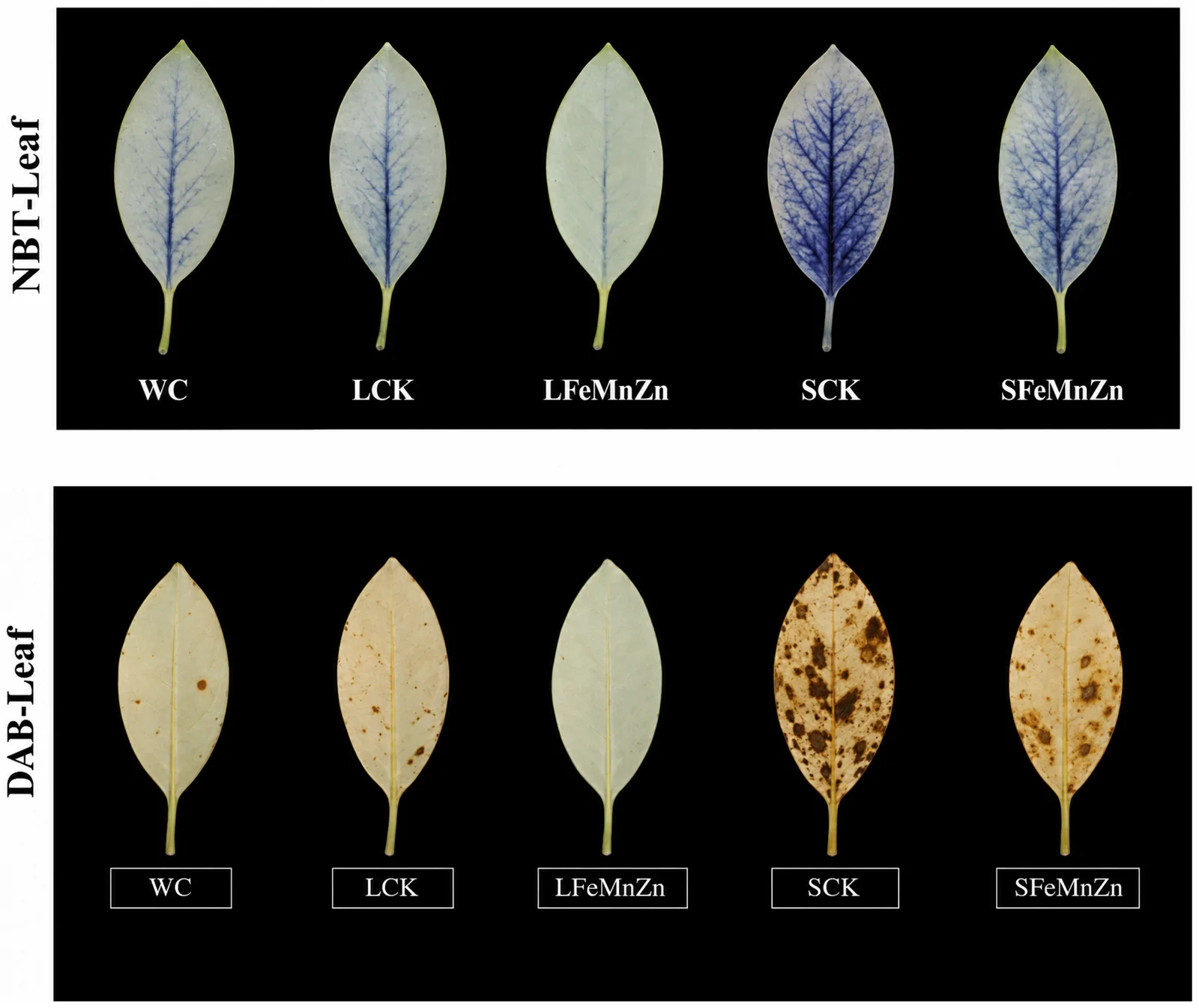
Representative leaf NBT staining (**upper**) and DAB staining (**lower**) for WC, LCK, LFeMnZn, SCK and SFeMnZn. Blue NBT and brown DAB staining provide qualitative observations associated with superoxide and H_2_O_2_, respectively. Original image content and treatment order are retained. Statistical conclusions derive from the independent pot-level biochemical measurements, not image color intensity.

Combined supplementation decreased H_2_O_2_ concentrations by 37.6% in leaves and 43.9% in roots, and superoxide production by 47.6% and 48.3%, relative to SCK (Figure 5; all adjusted *p* < 0.05). MDA decreased by 33.9% and 37.9%, while REC decreased by 40.5% and 39.0% (all adjusted *p* < 0.01). Thus, lower ROS measures coincided with less lipid peroxidation and electrolyte leakage in both organs. Representative leaves showed stronger NBT-associated blue staining and DAB-associated brown staining in SCK than in SFeMnZn (Figure 6), consistent with the directions of the superoxide and H_2_O_2_ measurements. These images provide qualitative support; significance is based on the independent pot-level assays. Root fluorescence images provide additional qualitative observations (Figure S2).

### 3.6. Antioxidant and Phenolic-Metabolism Responses

**Figure 7:**
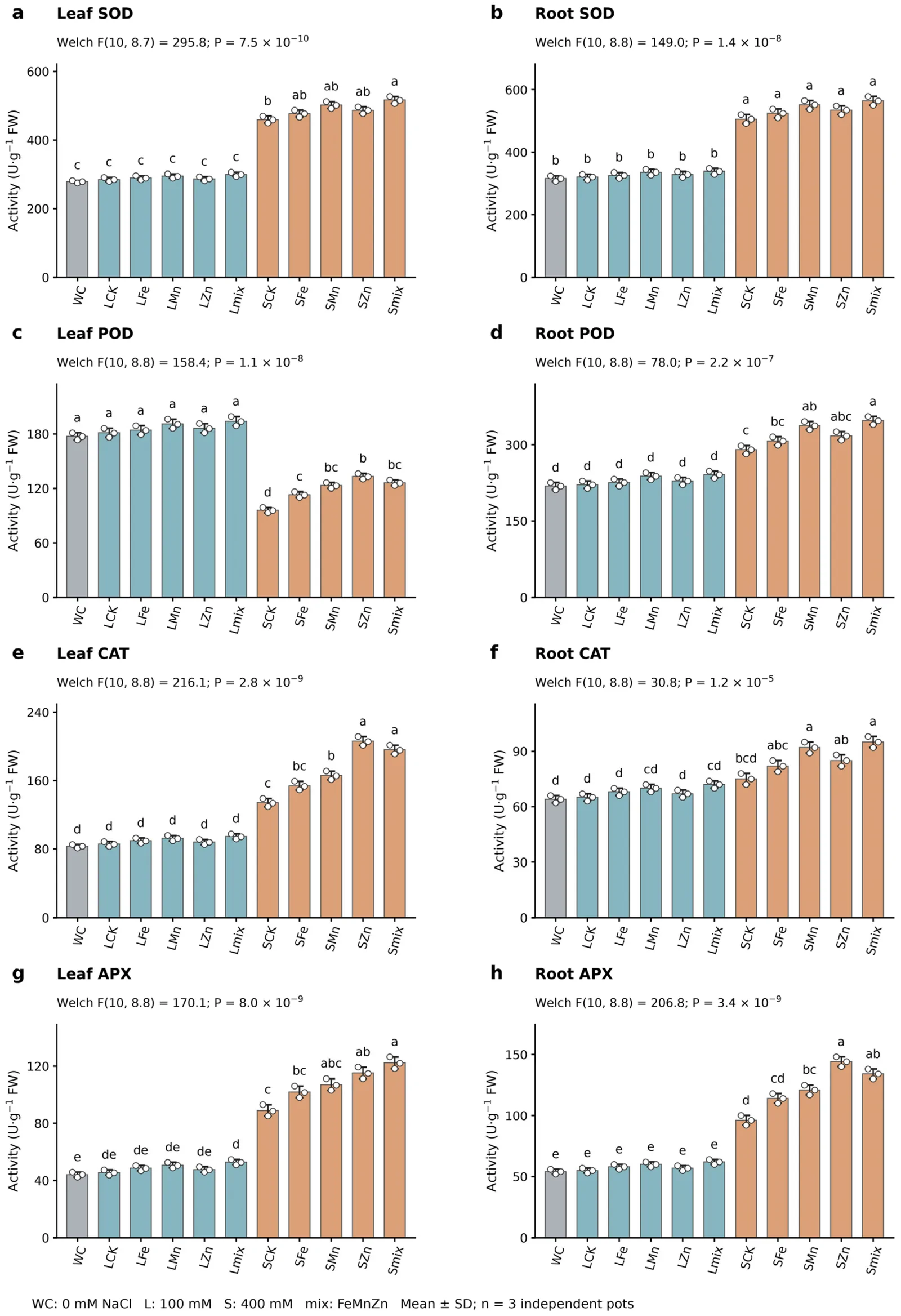
Leaf and root superoxide dismutase (SOD), peroxidase (POD), catalase (CAT) and ascorbate peroxidase (APX) activities. (**a**) Leaf SOD; (**b**) root SOD; (**c**) leaf POD; (**d**) root POD; (**e**) leaf CAT; (**f**) root CAT; (**g**) leaf APX; (**h**) root APX. Bars show means ± SD; points represent three independent pots. Welch ANOVA results are shown. Letters are compared only within each endpoint using Games–Howell tests (α = 0.05); groups with no shared letter differ significantly. WC, 0 mM NaCl; L, 100 mM; S, 400 mM; Lmix and Smix, LFeMnZn and SFeMnZn. All panels have continuous zero-based axes.

Relative to SCK, SFeMnZn increased leaf POD, CAT and APX activities by 31.3%, 46.3% and 37.4%, and root activities by 19.7%, 26.7% and 39.6% (all adjusted *p* < 0.05; Figure 7). Leaf SOD increased by 12.4% (*p* = 0.0245), whereas root SOD did not differ significantly (*p* = 0.0727). Leaf POD remained below the WC level despite its increase relative to SCK.

**Figure 8:**
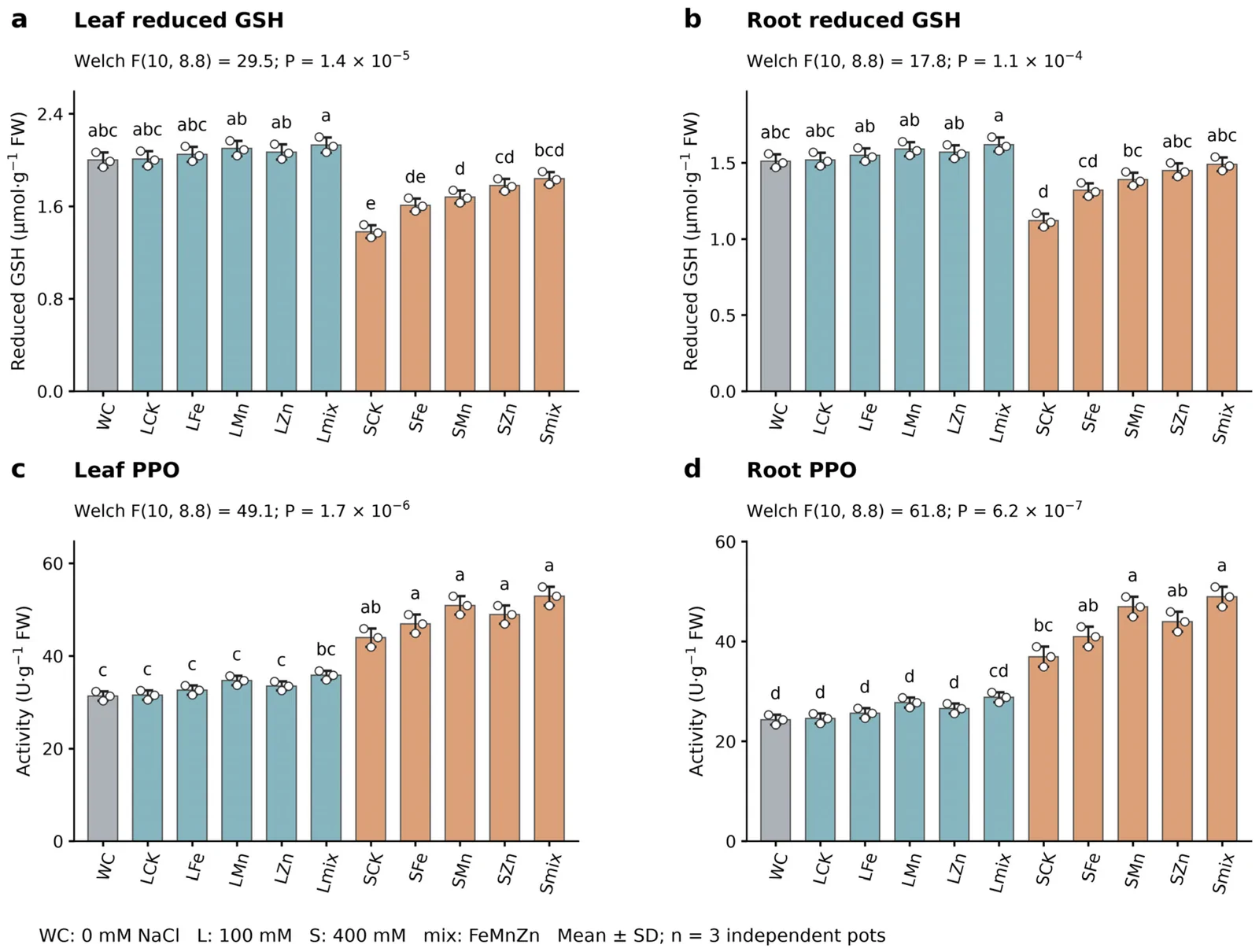
Reduced glutathione (GSH) concentrations and polyphenol oxidase (PPO) activities in leaves and roots. (**a**) Leaf GSH; (**b**) root GSH; (**c**) leaf PPO; (**d**) root PPO. Bars show means ± SD; points represent three independent pots. Welch ANOVA results are shown. Letters are compared only within each endpoint using Games–Howell tests (α = 0.05); groups with no shared letter differ significantly. WC, 0 mM NaCl; L, 100 mM; S, 400 mM; Lmix and Smix, LFeMnZn and SFeMnZn. All panels have continuous zero-based axes.

Reduced GSH increased by 33.3% in leaves and 33.0% in roots (adjusted *p* = 0.0061 and 0.0067; Figure 8). Root PPO increased by 32.5% (*p* = 0.0201); the leaf PPO increase was not significant (*p* = 0.0552). None of these 12 LFeMnZn–LCK comparisons reached significance.

### 3.7. Leaf Transcriptional Responses to Combined Supplementation

**Figure 9:**
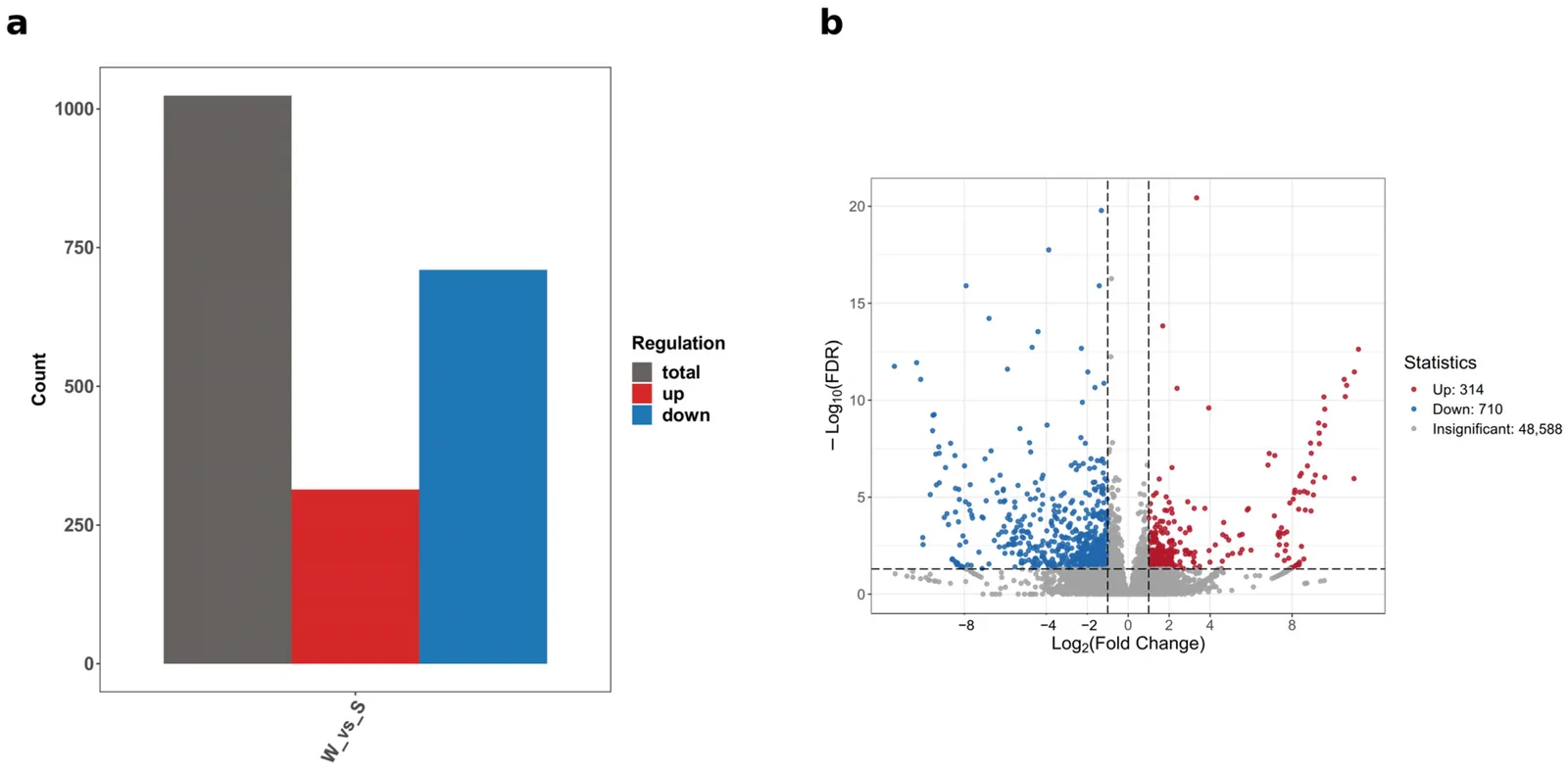
Report-derived differential-expression overview. (**a**) Numbers of total, upregulated and downregulated differentially expressed entries. (**b**) Volcano plot of log2 fold change versus −log10(FDR). The vertical dashed lines mark log2 fold changes of −1 and +1; the horizontal dashed line marks FDR = 0.05. Positive log2 fold changes indicate higher expression in SFeMnZn than in SCK. S denotes SCK and W denotes SFeMnZn, both under the initial 400 mM NaCl treatment. The comparison identified 314 upregulated and 710 downregulated entries under combined supplementation; the original service figure is retained.

**Figure 10:**
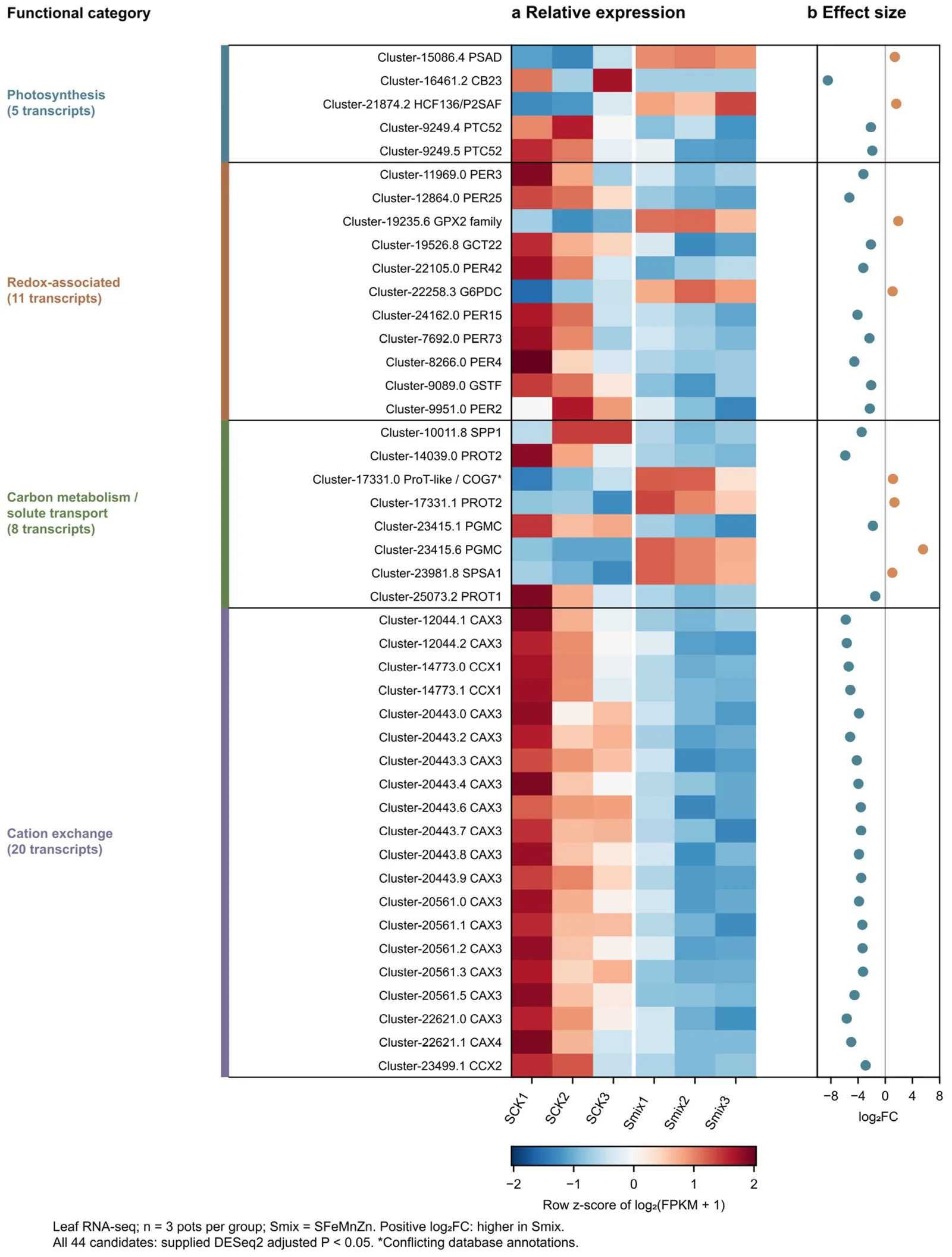
Annotation-selected leaf transcript candidates. (**a**) Row z-scores of log_2_(FPKM + 1); (**b**) supplied log_2_ fold changes for SFeMnZn relative to SCK. Colored sidebars indicate annotation-based functional categories, and black horizontal lines delimit their entries. Each Cluster row is aligned across the heatmap and effect-size plot; counts in parentheses indicate displayed entries. Groups were defined by functional annotations, not expression clustering. All 44 entries meet the supplied DESeq2 thresholds; the asterisk marks conflicting NR and Swiss-Prot annotations. HCF136 and P2SAF designate the same candidate entry, Cluster-21874.2, in this figure and do not represent two genes.

The six libraries yielded 59.4 Gb of clean data, with Q30 of at least 97%, and assembly produced 74,240 unigenes. Within-treatment Pearson correlations ranged from 0.980 to 0.988 for SCK and from 0.980 to 0.989 for SFeMnZn, compared with 0.914–0.933 between treatments (Figure S3). PC1 and PC2 accounted for 75.98% and 11.82% of the variance, respectively (87.80% combined). The two treatments occupied separate regions along PC1. The comparison identified 1024 differentially expressed entries, including 314 upregulated and 710 downregulated entries (Figure 9). The annotation screen retained 44 candidates (Figure 10). Candidates annotated as photosystem I reaction center subunit II (PSAD) and the photosystem II assembly factor HIGH CHLOROPHYLL FLUORESCENCE 136 (HCF136) increased (Cluster-15086.4 and Cluster-21874.2, the latter labeled HCF136/P2SAF in Figure 10; log_2_FC = 1.409 and 1.619). Candidates annotated as chlorophyll a/b-binding protein and protochlorophyllide-dependent translocon components decreased. These annotations link the candidates to light harvesting and photosynthetic apparatus assembly.

Candidates annotated as glutathione peroxidase 2-family protein (GPX2) and glucose-6-phosphate dehydrogenase (G6PD), Cluster-19235.6 and Cluster-22258.3, increased (log_2_FC = 1.916 and 1.081). Glutathione S-transferase (GST), gamma-glutamylcyclotransferase (GGCT) and seven peroxidase candidates decreased. These proteins are associated with peroxide metabolism, glutathione-related processes and reductant supply. Sucrose-phosphate synthase and sucrose-phosphatase candidates, involved in sucrose synthesis, showed opposite responses; two phosphoglucomutase entries also differed in direction. Proline transporter (ProT)-related candidates changed in both directions, and Cluster-17331.0 retained its flag for conflicting NR and Swiss-Prot annotations. All 20 screened cation-exchanger entries, including cation/proton exchanger (CAX) and cation/calcium exchanger (CCX) candidates, were downregulated.

**Figure 11:**
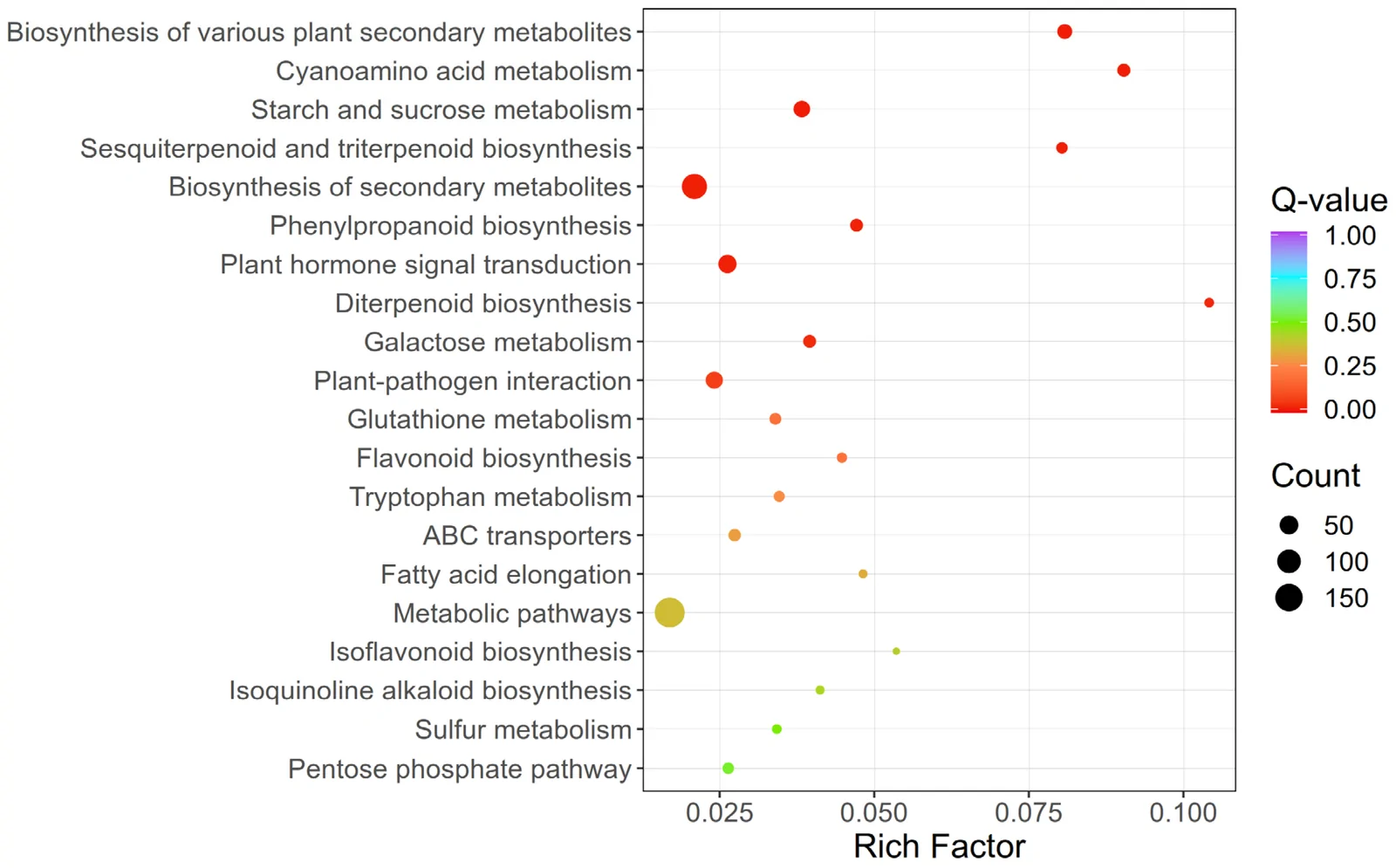
KEGG enrichment display from the service report. Faint background guides were added without changing bubble positions, sizes or colors. The reported rich factors, count legend and q-value scale are retained; not every displayed pathway is necessarily significant at q < 0.05.

The reported KEGG display included secondary-metabolite biosynthesis, starch and sucrose metabolism, phenylpropanoid biosynthesis and glutathione metabolism (Figure 11). The GO chord diagram linked representative candidates with ion transport and homeostasis terms (Figure S1). Cellular zinc homeostasis, cellular manganese homeostasis, manganese homeostasis and phosphate homeostasis contained 16, 16, 17 and 18 of the 723 GO-annotated DEGs, respectively. These categories overlap. The reported pathway displays provide functional context; the presence of a pathway in a top-ranked display alone was not used to assign significance.

## 4. Discussion

### 4.1. Combined Supplementation Improves Tissue Nutrition, Root Development and Photosynthetic Growth

Combined Fe, Mn and Zn supplementation increased the corresponding elemental concentrations in leaves and roots under the high-salt treatment, together with improvements in root architecture, photosynthetic performance and biomass. Total root length, surface area and volume increased in SFeMnZn relative to SCK, accompanied by more root tips and forks. Longer, more branched root systems can increase contact with the growth substrate, providing a structural basis for water and nutrient acquisition. The concurrent changes in tissue elemental concentrations and root growth show that the treatment response involved both the plant’s nutritional status and below-ground development.

Leaf gas exchange, pigment concentrations and chlorophyll fluorescence link this growth response to improved photosynthetic function. Higher stomatal conductance and intercellular CO_2_ in SFeMnZn are consistent with improved CO_2_ supply, while the increases in the net photosynthetic rate, chlorophyll and Fv/Fm indicate better carbon assimilation and photochemical performance. A comparable nutritional benefit was reported in pak choi, where foliar Zn improved water relations and alleviated growth inhibition under salt treatment [11]. This agrees with the growth improvements observed here under Zn and combined supplementation.

Studies of halophytes also connect photosynthetic performance with growth. Lu et al. found that enhanced growth of *Nitraria sibirica* under salt treatment accompanied higher photosynthetic performance and altered expression of carbon-fixation genes [38]. Here, nutrient supplementation produced concurrent improvements in photosynthesis and biomass, supporting the importance of maintaining leaf carbon acquisition for seedling growth in saline conditions.

Among the individual high-salt supplements, SZn had the highest mean values for the measured growth traits, and several root traits were significantly higher than in SFe and SMn. Dry mass did not differ significantly among the three individual supplements. Zn therefore warrants particular attention in future formulation optimization. Combined supplementation increased Fe, Mn and Zn concentrations in both organs and was associated with improved root development, leaf photosynthesis and dry-matter production, providing a basis for integrated nutritional management of *R. stylosa* seedlings.

### 4.2. Antioxidant Adjustment and Reduced Membrane Injury Accompany Improved Seedling Growth

Combined supplementation lowered H_2_O_2_ concentrations, superoxide production, MDA concentrations and relative electrolyte conductivity in leaves and roots. Lower ROS measures indicate reduced oxidative burden, lower MDA is consistent with less membrane lipid peroxidation, and lower electrolyte leakage indicates improved membrane integrity. Agreement among these measurements in both organs supports a protective physiological response accompanying improved growth.

Higher POD, CAT and APX activities provide a physiological explanation for improved peroxide removal. CAT decomposes H_2_O_2_, APX uses ascorbate to remove H_2_O_2_, and POD also participates in peroxide metabolism. Reduced GSH concentrations increased in leaves and roots, indicating responses in both enzymatic defense and nonenzymatic antioxidant pools. Zhou et al. linked salinity responses in *Sonneratia caseolaris* to POD, CAT, APX and glutathione-related genes [12], while the *Aegiceras corniculatum* study by Feng et al. linked antioxidant processes to cellular homeostasis in saline habitats [39]. The antioxidant changes and reduced injury observed here support maintenance of redox balance as an important component of the supplementation response.

Soluble sugar and proline concentrations also decreased in leaves and roots. Together with improved photosynthesis, dry mass and membrane integrity, these changes are consistent with reduced demand for stress-associated solute accumulation and greater use of assimilated carbon for growth. Lower sugar and proline concentrations should therefore be interpreted alongside better growth and less tissue injury. The combined measurements suggest a shift in allocation between growth and stress responses; the concentration of a single solute alone would not capture this adjustment.

Polyphenol oxidase (PPO) oxidizes phenolic compounds to quinones and participates in plant secondary metabolism. Combined supplementation increased root PPO activity by 32.5%, alongside higher root GSH, increased activities of several antioxidant enzymes, and lower MDA and electrolyte leakage. Araji et al. showed that PPO silencing in walnut altered phenolic and tyrosine-derivedmetabolism and caused spontaneous leaf necrosis, linking PPO to normal tissue metabolism [40]. A useful next step is to identify the root phenolics and oxidation products that change after supplementation and relate them to PPO activity, membrane injury and root-tip growth. Such work could test whether PPO-associated metabolism contributes to root protection and connect the enzyme response with specific metabolites and growth traits.

### 4.3. Leaf Transcriptional Responses Identify Candidates for Photosystem Maintenance, Peroxide Metabolism and Resource Allocation

The SFeMnZn–SCK leaf transcriptome comparison connects nutrient supplementation under high salinity with specific molecular processes. The selected candidates involve photosystems, redox metabolism, sugar and compatible-solute transport, and cation exchange. These processes correspond to the measured changes in photosynthesis, antioxidant indicators and tissue elements, extending the physiological observations to candidate metabolic responses within leaves.

Candidates annotated as photosystem I reaction center subunit II (PSAD) and the photosystem II assembly factor HIGH CHLOROPHYLL FLUORESCENCE 136 (HCF136) were upregulated. PSAD is a component of photosystem I, whereas HCF136 participates in photosystem II assembly and stability. Meurer et al. showed that loss of HCF136 function in Arabidopsis severely impaired photosystem II stability and activity [41]. Here, higher expression of the HCF136 candidate accompanied increased Fv/Fm and the net photosynthetic rate, suggesting that maintaining photosystem assembly and function may contribute to the photosynthetic benefit of supplementation.

Redox-related candidates provide a further connection with antioxidant responses. Glucose-6-phosphate dehydrogenase (G6PD) generates NADPH through the oxidative pentose phosphate pathway, supplying reducing power for cellular antioxidant systems. Yang et al. found that loss of cytosolic G6PD function in Arabidopsis reduced NADPH and GSH under salt treatment and aggravated oxidative injury and root-growth inhibition, whereas increased expression supported antioxidant capacity [42]. Upregulation of the leaf G6PD candidate here accompanied higher GSH and lower ROS, consistent with this physiological connection. Sustaining reducing-power supply may therefore help explain reduced leaf oxidative injury. Sulfur supplementation also increased GSH and improved photosynthesis in salt-treated mustard [43], suggesting that sulfur supplied with the supplemental salts may contribute to these responses and warrant investigation alongside sulfur assimilation and GSH metabolism.

The candidate annotated as glutathione peroxidase 2-family protein (GPX2) was also upregulated, identifying a further response in peroxide metabolism. Selected glutathione S-transferase (GST), gamma-glutamylcyclotransferase (GGCT) and peroxidase candidates were downregulated. Different members of an enzyme family can show opposing responses: Zhou et al. reported two upregulated and three downregulated POD-related genes in their *Sonneratia caseolaris* salinity study [12]. Our comparison describes the supplementation response after 90 days. In the context of lower ROS and tissue injury, reduced expression of some stress-associated entries may reflect a lower oxidative burden, while candidates involved in reducing-power supply and peroxide metabolism remain responsive.

The POD assay measures the combined activity of multiple peroxidase proteins, whereas each transcript entry represents an individual annotated sequence. Integrated transcriptomic and proteomic analysis of salt-treated Arabidopsis by Feng et al. showed that transcript and protein-abundance changes were not always concordant [44]. Downregulation of selected peroxidase candidates can therefore coexist with increased total POD activity. Considered together, enzyme activities, GSH and ROS measurements support enhanced antioxidant protection and reduced oxidative injury under combined supplementation.

Sugar metabolism and solute transport candidates provide further clues for the sugar and proline responses. Sucrose-phosphate synthase (SPS) and sucrose-phosphatase (SPP) catalyze successive steps in sucrose synthesis, while phosphoglucomutase (PGM) interconverts glucose-1-phosphate and glucose-6-phosphate, linking sugar synthesis and utilization. Their expression changes, together with higher net photosynthesis and lower soluble sugar concentrations, suggest altered allocation of photosynthetic carbon between accumulation and growth. Changes in proline transporter (ProT)-related candidates also point to solute redistribution. Functional characterization of the Arabidopsis ProT family demonstrated transport of proline and other compatible solutes [45], providing a basis for examining the connection between proline concentrations and transport-related expression in this study.

Cation-exchange candidates identify a further response associated with intracellular ion distribution. All screened cation/proton exchanger (CAX) and cation/calcium exchanger (CCX) entries were downregulated, while leaf and root Fe, Mn and Zn concentrations increased. CAX proteins contribute to intracellular cation distribution and storage and to maintenance of an appropriate cellular ionic environment [46]. Combining tissue elemental measurements with these expression responses identifies a specific question for further work: how are supplemented elements distributed and used within plant cells? The CAX and CCX candidates provide targets for investigating that process.

Together, the leaf transcriptional responses connect the physiological changes with photosystem maintenance, reducing power supply and peroxide metabolism, and the allocation of sugars, compatible solutes and cations. These connections provide molecular leads for understanding the concurrent improvements in photosynthesis, tissue integrity and growth.

### 4.4. Greenhouse Nursery Applications and Future Research

This greenhouse sand-culture study provides a basis for evaluating the supplementation regime in controlled *R. stylosa* nursery production and pre-transplant conditioning. Temperature, nutrient-solution composition and application frequency can be managed in a greenhouse. Temperature is relevant because it modifies *R. stylosa* salinity responses [2]. More extensive roots, higher photosynthetic performance and lower tissue injury are useful criteria for selecting nursery treatments. Future work could optimize concentration, application frequency and root versus foliar delivery to retain growth benefits while controlling nutrient inputs.

The intended application is the production of seedlings suitable for transplantation. After coastal transplantation, tidal exchange changes root-zone water and nutrient conditions. Future studies could therefore assess whether the advantages developed during greenhouse cultivation persist in post-transplant survival, growth and root establishment. Following seedlings from nursery treatment through field establishment would clarify the practical value of the regime for mangrove planting-material production.

The present comparisons establish the responses to individual and combined sulfate supplements under saline cultivation. Future studies incorporating the same supplements without added NaCl could distinguish general nutritional benefits from responses that are specific to salt exposure. Sulfate-matched controls were not included, so the findings support the overall effect of the tested formulation; the separate contributions of the metals and accompanying sulfur, and any synergy among the metals, remain to be resolved. Future comparisons could include two-element mixtures and balanced sulfur and other accompanying-ion inputs, together with tissue sulfur, cysteine and GSH measurements, to distinguish the contributions to growth and antioxidant protection. Tracking root-zone salinity during cultivation would also relate the initial solution treatments more closely to the subsequent salt exposure.

Molecular studies could prioritize G6PD, HCF136 and CAX/CCX candidates and connect their expression with enzyme activity, protein localization or ion-transport function. These investigations would help to translate the candidate processes identified here into a more specific basis for formulation optimization.

## 5. Conclusions

Under the greenhouse conditions tested, combined Fe, Mn and Zn sulfate supplementation increased *R. stylosa* seedling dry mass, total root length and net photosynthetic rate by 29.8%, 133.1% and 67.0%, respectively, relative to SCK. Improved growth was accompanied by higher leaf and root micronutrient concentrations, lower ROS and membrane injury, and changes in antioxidant and osmolyte responses. Upregulated leaf candidates annotated as PSAD, HCF136, G6PD and GPX2 provide molecular leads for photosystem maintenance, reducing-power supply and peroxide metabolism, and identify targets for investigating the links between improved photosynthesis and reduced oxidative injury. The tested formulation has potential for improving seedling quality under high-salt cultivation and provides a basis for further development of greenhouse nursery and pre-transplant nutrient management.

## Figures and Tables

**Table 1:** Experimental treatments and supplemental working-solution concentrations.

Treatment	Initial NaCl (mM)	FeSO_4_·7H_2_O (mg·L^−1^)	MnSO_4_·H_2_O (mg·L^−1^)	ZnSO_4_·H_2_O (mg·L^−1^)
**WC**	0	0	0	0
**LCK**	100	0	0	0
**LFe**	100	150	0	0
**LMn**	100	0	312.5	0
**LZn**	100	0	0	138.9
**LFeMnZn**	100	150	312.5	138.9
**SCK**	400	0	0	0
**SFe**	400	150	0	0
**SMn**	400	0	312.5	0
**SZn**	400	0	0	138.9
**SFeMnZn**	400	150	312.5	138.9

All groups received quarter-strength Hoagland basal nutrient solution. Zero denotes no additional sulfate supplement. Each treatment comprised three independent pots with five seedlings per pot.

**Table 2:** Leaf and root Fe, Mn and Zn concentrations.

Treatment	Leaf Fe	Leaf Mn	Leaf Zn	Root Fe	Root Mn	Root Zn
WC	100.80 ± 2.07 c	61.47 ± 1.50 cd	29.63 ± 0.86 b	178.60 ± 3.87 b	118.40 ± 3.83 b	42.60 ± 1.28 c
LCK	99.13 ± 2.11 c	60.70 ± 1.21 cd	29.07 ± 0.75 b	176.90 ± 3.61 b	116.70 ± 3.51 b	41.80 ± 1.30 c
LFe	145.47 ± 4.19 b	62.37 ± 1.56 cd	30.57 ± 1.31 bc	284.67 ± 5.95 a	121.57 ± 3.71 b	45.20 ± 2.29 c
LMn	100.57 ± 2.75 c	142.63 ± 6.68 b	30.07 ± 1.15 b	181.50 ± 4.42 b	265.33 ± 8.71 a	43.50 ± 1.48 c
LZn	101.40 ± 2.49 c	63.03 ± 1.68 c	81.80 ± 4.62 a	183.40 ± 4.25 b	123.50 ± 4.27 b	126.70 ± 4.75 ab
LFeMnZn	155.93 ± 4.12 ab	148.67 ± 6.35 ab	87.17 ± 4.61 a	305.80 ± 7.40 a	279.60 ± 10.59 a	138.40 ± 5.68 a
SCK	84.60 ± 2.02 d	52.90 ± 1.41 e	24.80 ± 0.70 c	150.80 ± 4.23 c	91.50 ± 3.16 c	34.50 ± 1.28 d
SFe	151.40 ± 4.67 ab	54.47 ± 1.96 de	26.57 ± 0.91 bc	271.60 ± 7.69 a	96.80 ± 3.16 c	36.80 ± 1.47 cd
SMn	86.47 ± 2.62 d	174.50 ± 7.55 ab	26.10 ± 0.95 bc	158.70 ± 3.99 c	248.90 ± 9.60 a	38.20 ± 1.61 cd
SZn	87.40 ± 2.59 d	55.30 ± 2.00 cde	72.93 ± 3.58 a	154.90 ± 3.82 c	98.40 ± 3.46 c	108.50 ± 4.48 b
SFeMnZn	167.83 ± 4.23 a	181.63 ± 7.72 a	81.03 ± 4.27 a	296.50 ± 7.75 a	270.40 ± 10.62 a	121.70 ± 4.59 ab

Values are mean ± SD (mg·kg^−1^ DW), n = 3 independent pots. Letters are compared within each column only using Games–Howell tests following Welch ANOVA (α = 0.05). Groups without a common letter differ significantly; shared letters indicate no detected difference. All treatments received basal nutrient solution; CK indicates no additional Fe/Mn/Zn supplementation.

## Data Availability

The raw RNA-seq reads have been deposited in the NCBI Sequence Read Archive under BioProject accession PRJNA1538750 (https://www.ncbi.nlm.nih.gov/bioproject/PRJNA1538750, accessed on 21 October 2026), with run accessions SRR41004775–SRR41004780. Other data supporting the findings of this study are available from the corresponding author upon request.

## Supplementary Materials

The following supporting information can be downloaded at https://www.mdpi.com/article/10.3390/biology15211933/s1: Supplementary Methods S1: Detailed biochemical assays and oxidative-stress imaging procedures; Supplementary Methods S2: Treatment selection and application; Figure S1: GO associations from the service report; Figure S2: Representative root CM-H2DCFDA fluorescence images; Figure S3: Sample correlations and principal component analysis of SCK and Smix (SFeMnZn). References [3,13,14,15,16,20,21,22,23,24,25,26,27,28,29,30,31,32,33] are cited in the Supplementary Materials.
