# Supplementary material for: Combined Supplementation with Iron, Manganese and Zinc Sulfates Improves Growth and Modulates Leaf Gene Expression in Salt-Stressed Rhizophora stylosa Seedlings
Source: Biology (Basel). 2026 Oct 24;15(21):1933. doi: 10.3390/biology15211933 (PMC13762278; doi:10.3390/biology15211933)
Supplement: Supplementary file 1 [file biology-15-01933-s001.zip › biology-4639516-supplementary.pdf]

Supplementary Materials

Combined supplementation with iron, manganese and zinc sulfates improves growth and modulates leaf gene expression in salt-stressed Rhizophora stylosa seedlings

Supplementary Methods S1

Detailed biochemical assays and oxidative-stress imaging procedures

Leaf and root samples for elemental analysis were heated at 105 °C for 30 min and then dried at 75 °C to constant mass, using the same relative mass-change criterion (<0.1%). Dried samples were ground and sieved. A 0.2000 g aliquot of leaf or root tissue was placed in a PTFE digestion vessel with 5.0 mL concentrated HNO_3 and 1.0 mL 30% H_2O_2 (5:1, v/v). After approximately 30 min of predigestion at room temperature, samples underwent closed-vessel microwave digestion. NIST SRM 1573a (Tomato Leaves) was included as a reference material for analytical quality control [S1]. Fe, Mn and Zn were measured separately by inductively coupled plasma mass spectrometry (Agilent 7500cx, Agilent Technologies, USA), with concentrations expressed as mg kg^{-1} dry weight (DW).

Soluble sugars were determined by anthrone colorimetry [S2]. Proline was extracted using 3% sulfosalicylic acid and measured by the acid-ninhydrin method with glacial acetic acid and toluene at 520 nm [S3], expressed as μg g^{-1} fresh weight (FW). Reduced glutathione (GSH) was extracted with 5% sulfosalicylic acid and assayed with DTNB at 412 nm [S4], expressed as μmol g^{-1} FW.

For superoxide dismutase (SOD), peroxidase (POD) and catalase (CAT) assays, 0.50 g of fresh leaf or root tissue was homogenized on ice with 5.0 mL of cold 50 mM potassium phosphate buffer (pH 7.0) containing 1 mM EDTA and 1% PVP. Homogenates were centrifuged at approximately 12,000 × g for 15–20 min at 4 °C, and the supernatant was used as the enzyme extract. Ascorbate peroxidase (APX) was extracted separately with 1 mM ascorbate added to the extraction buffer.

SOD activity was measured by inhibition of NBT photoreduction [S5] in a 3.0 mL reaction containing 50 mM phosphate buffer (pH 7.8), 13 mM methionine, 75 μM NBT, 0.1 mM EDTA, 2 μM riboflavin and enzyme extract. After approximately 15 min of illumination, absorbance was measured at 560 nm, with illuminated and dark enzyme-free controls. One unit was defined as the amount of enzyme producing 50% inhibition of NBT photoreduction.

POD was assayed using guaiacol oxidation [S6] at 25 °C in a 3.0 mL reaction containing 50 mM phosphate buffer (pH 6.5–7.0), 20 mM guaiacol, 10 mM H_2O_2 and enzyme extract. The increase in absorbance at 470 nm was recorded for approximately 60 s; the tetraguaiacol extinction coefficient was 26.6 mM^{-1} cm^{-1}. CAT was assayed spectrophotometrically [S7] in a 3.0 mL reaction containing 50 mM phosphate buffer (pH 7.0), 10 mM H_2O_2 and enzyme extract by monitoring the decrease at 240 nm for 1 min. The H_2O_2 extinction coefficient was 39.4 M^{-1} cm^{-1} (0.0394 mM^{-1} cm^{-1}). APX was assayed using ascorbate oxidation [S8] with 50 mM phosphate buffer (pH 7.0), 0.5 mM ascorbate, 0.1 mM EDTA, 0.1 mM H_2O_2 and APX extract. The decrease at 290 nm was recorded for approximately 1 min using an ascorbate extinction coefficient of 2.8 mM^{-1} cm^{-1}. One unit of POD, CAT or APX represented formation of 1 μmol tetraguaiacol, decomposition of 1 μmol H_2O_2 or oxidation of 1 μmol ascorbate per minute, respectively. PPO was assayed by catechol oxidation at 420 nm [S9]; one unit was defined in this study as an increase in A420 of 0.01 min^{-1}. Enzyme activities were expressed as U g^{-1} FW according to the assay-specific unit definitions.

Malondialdehyde (MDA) was determined using a modified TBA–TCA lipid-peroxidation assay [S10] using absorbance at 450, 532 and 600 nm. H_2O_2 was measured by KI colorimetry at 390 nm [S11], and superoxide production by hydroxylamine oxidation at 530 nm [S12]. MDA was expressed as nmol g^{-1} FW and H_2O_2 as μmol g^{-1} FW, and superoxide production as nmol g^{-1} FW min^{-1}. Relative electrolyte conductivity (REC) was measured by the conductivity method [S10] and expressed as a percentage. Leaf staining procedures were adapted from histochemical detection methods [S13]. For leaf DAB staining, fully expanded leaves of

comparable developmental stage were vacuum-infiltrated for approximately 5 min and incubated with 1 mg mL^{-1} DAB (pH 3.8) at 25 °C in darkness for 8 h. For NBT staining, leaves were vacuum-infiltrated for 2–5 min with 0.1% (w/v) NBT in 50 mM phosphate buffer (pH 7.8) containing 10 mM NaN_3 and incubated at 25 °C in darkness for 2 h. Leaves were decolorized with 95–96% ethanol before photography. Leaf position, staining and decolorization times, illumination, white balance and exposure were kept consistent within each staining assay. The images were used as qualitative supporting observations.

Root oxidative activity was visualized using CM-H_2DCFDA [S14]. Fresh root tips, approximately 1–2 cm long, were rinsed with buffer and incubated with 10 µM CM-H_2DCFDA at 25 °C in darkness for 20 min. They were rinsed three times with buffer for approximately 3–5 min each and imaged immediately. Root fluorescence was observed with a Leica M165 FC fluorescence stereomicroscope using the GFP plants filter set (excitation 450–490 nm; emission 500–550 nm). Images were collected at an exposure time of 150 ms, gain of 1.0× and gamma of 1.0, with consistent magnification and illumination across treatments. The image calibration was 200 µm per 130 source-image pixels (approximately 1.538 µm $pixel^{-1}$); scale bars were drawn using this calibration. CM-H_2DCFDA fluorescence was interpreted as a qualitative indicator of oxidative activity rather than a selective measurement of H_2O_2.

Supplementary Methods S2

Treatment selection and application

Pre-experimental field measurements in the Jinniu Island mangrove waters indicated salinities of approximately 3–25‰. The initial 100 and 400 mM NaCl solution treatments were selected considering these observations, the regional salinity background [S15], published mangrove cultivation studies [S16, S17], and seedling growth in preliminary cultivation. They correspond to approximately 5.84 and 23.38 g L^{-1} NaCl. These values describe the applied NaCl solutions; natural-water salinity represents mixed salts.

The micronutrient working concentrations were selected using preliminary observations of normal seedling growth and published studies of ferrous sulfate and manganese sulfate foliar supplementation [S18, S19]. Solutions contained 150 mg L^{-1} $FeSO_4 \cdot 7H_2O$, 312.5 mg L^{-1} $MnSO_4 \cdot H_2O$ and/or 138.9 mg L^{-1} $ZnSO_4 \cdot H_2O$, approximately equivalent to 30, 100 and 50 mg L^{-1} of Fe, Mn and Zn, respectively. The mixture contained all three salts at these concentrations. Each pot received 100 mL as a root drench and 100 mL as a foliar spray, with 20 mL sprayed per seedling. Twelve applications were made at seven-day intervals on days 7–84 of the 90-day formal treatment period. Low- and high-salt groups received their respective preprepared 100 and 400 mM NaCl solutions directly at the start of treatment.

Each treatment comprised three independent pots with five seedlings per pot. Three randomly selected seedlings supplied pooled tissue samples and root-scan measurements; their mean root measurements represented the pot. The remaining two intact seedlings supplied whole-plant dry mass. Plant height, basal stem circumference and gas-exchange measurements were averaged across all five seedlings. The pot was the unit of replication.

Figure S1

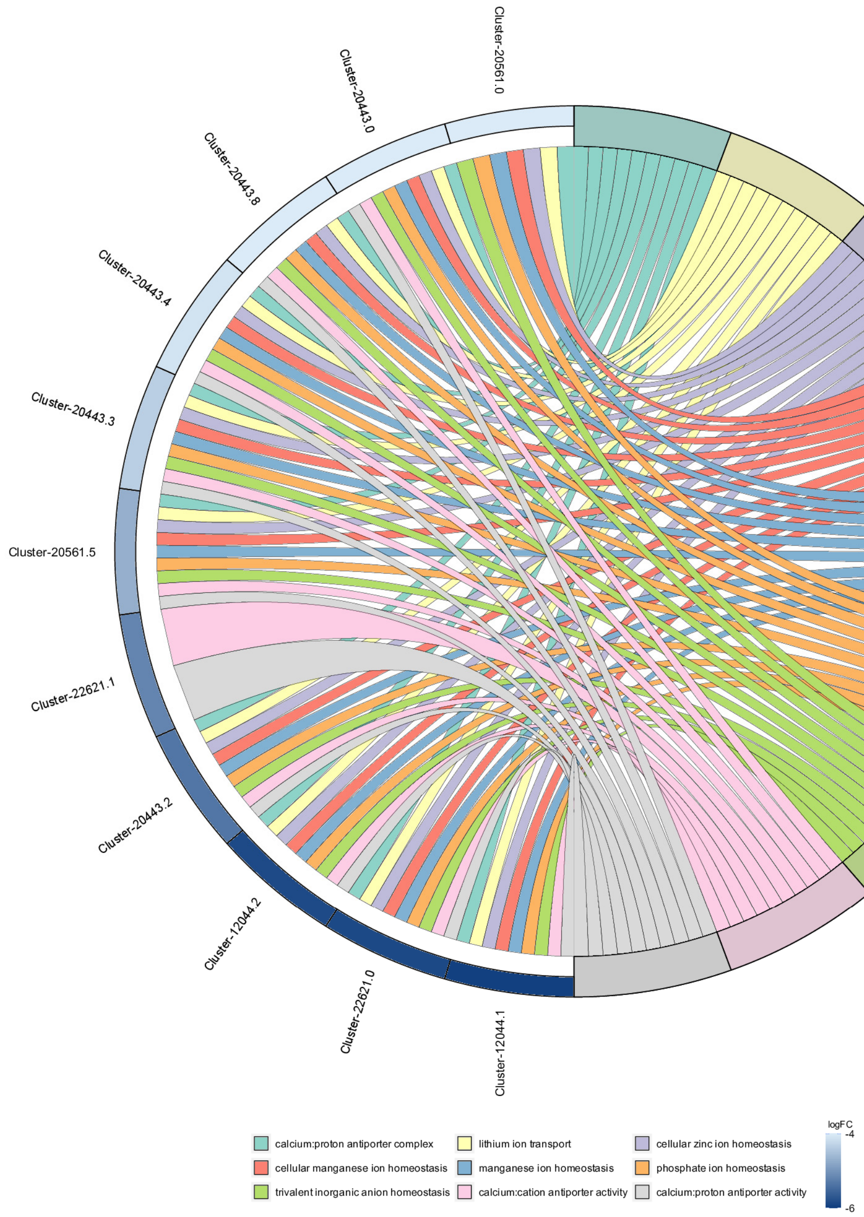

Figure S1. GO associations from the service report. Links connect gene entries and GO terms. The blue gene-ring colours and their matching logFC legend were remapped together for clearer contrast; topology, labels and values were preserved. S and W denote SCK and SFeMnZn.

Figure S2

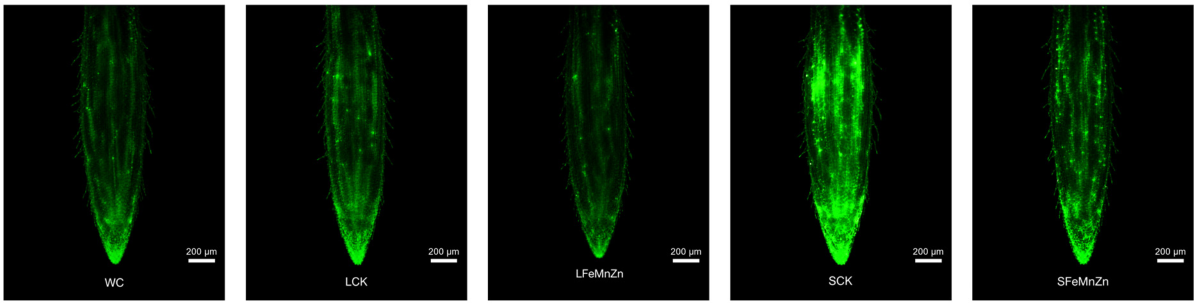

Figure S2. Representative root CM-H_2DCFDA fluorescence images showing qualitative differences in oxidative activity. Scale bars, 200 µm, correspond to 130 pixels in the source images (approximately 1.538 µm $pixel^{-1}$).

Figure S3

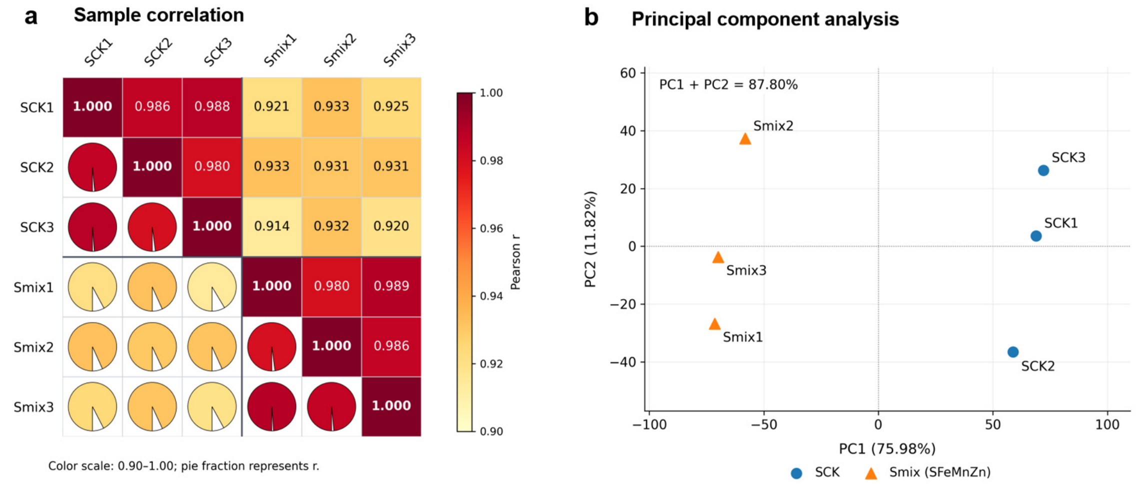

Figure S3. Sample correlations and principal component analysis of SCK and Smix (SFeMnZn), with three independent pot-level samples per treatment. Left: Pearson correlation coefficients; the colour scale spans 0.90–1.00 and pie fractions represent r. Right: PCA scores, with PC1 and PC2 explaining 75.98% and 11.82% of the variance (87.80% combined). Blue circles denote SCK and orange triangles denote Smix. Sample numbers identify biological replicates.

Supplementary references

S1. National Institute of Standards and Technology. Standard Reference Material 1573a: Tomato Leaves. Available online: https://tsapps.nist.gov/srmext/certificates/1573a.pdf (accessed on 17 October 2026).

S2. Yemm, E.W.; Willis, A.J. The estimation of carbohydrates in plant extracts by anthrone. Biochem. J. 1954, 57, 508–514. https://doi.org/10.1042/bj0570508.

S3. Bates, L.S.; Waldren, R.P.; Teare, I.D. Rapid determination of free proline for water-stress studies. Plant Soil 1973, 39, 205–207. https://doi.org/10.1007/BF00018060.

S4. Sedlak, J.; Lindsay, R.H. Estimation of total, protein-bound, and nonprotein sulfhydryl groups in tissue with Ellman's reagent. Anal. Biochem. 1968, 25, 192–205. https://doi.org/10.1016/0003-2697(68)90092-4.

S5. Giannopolitis, C.N.; Ries, S.K. Superoxide dismutases: I. Occurrence in higher plants. Plant Physiol. 1977, 59, 309–314. https://doi.org/10.1104/pp.59.2.309.

S6. Chance, B.; Maehly, A.C. Assay of catalases and peroxidases. Methods Enzymol. 1955, 2, 764–775. https://doi.org/10.1016/S0076-6879(55)02300-8.

S7. Aebi, H. Catalase in vitro. Methods Enzymol. 1984, 105, 121–126. https://doi.org/10.1016/S0076-6879(84)05016-3.

S8. Nakano, Y.; Asada, K. Hydrogen Peroxide is Scavenged by Ascorbate-specific Peroxidase in Spinach Chloroplasts. Plant Cell Physiol. 1981, 22, 867–880. https://doi.org/10.1093/oxfordjournals.pcp.a076232.

S9. Yuzuak, S.; Xie, D.-Y. An efficient protocol for the extraction of pigment-free active polyphenol oxidase and soluble proteins from plant cells. Biol. Methods Protoc. 2024, 9, bpae067. https://doi.org/10.1093/biomethods/bpae067.

S10. Jambunathan, N. Determination and detection of reactive oxygen species (ROS), lipid peroxidation, and electrolyte leakage in plants. Methods Mol. Biol. 2010, 639, 292–298. https://doi.org/10.1007/978-1-60761-702-0_18.

S11. Velikova, V.; Yordanov, I.; Edreva, A. Oxidative stress and some antioxidant systems in acid rain-treated bean plants: Protective role of exogenous polyamines. Plant Sci. 2000, 151, 59–66. https://doi.org/10.1016/S0168-9452(99)00197-1.

S12. Wang, A.G.; Luo, G.H. Quantitative relation between the reaction of hydroxylamine and superoxide anion radicals in plants. Plant Physiol. Commun. 1990, No. 6, 55–57. https://doi.org/10.13592/j.cnki.ppj.1990.06.031. (In Chinese)

S13. Kumar, D.; Yusuf, M.A.; Singh, P.; Sardar, M.; Sarin, N.B. Histochemical Detection of Superoxide and H_2O_2 Accumulation in Brassica juncea Seedlings. Bio-protocol 2014, 4, e1108. https://doi.org/10.21769/BioProtoc.1108.

S14. Bahmani, R.; Kim, D.; Na, J.; Hwang, S. Expression of the Tobacco Non-symbiotic Class 1 Hemoglobin Gene Hb1 Reduces Cadmium Levels by Modulating Cd Transporter Expression Through Decreasing Nitric Oxide and ROS Level in Arabidopsis. Front. Plant Sci. 2019, 10, 201. https://doi.org/10.3389/fpls.2019.00201.

S15. Liang, J.; Wright, J.S.; Cui, X.; Sternberg, L.; Gan, W.; Lin, G. Leaf anatomical traits determine the ^{18}O enrichment of leaf water in coastal halophytes. Plant Cell Environ. 2018, 41, 2744–2757. https://doi.org/10.1111/pce.13398.

S16. Parida, A.K.; Das, A.B.; Mittra, B. Effects of salt on growth, ion accumulation, photosynthesis and leaf anatomy of the mangrove, Bruguiera parviflora. Trees 2004, 18, 167–174. https://doi.org/10.1007/s00468-003-0293-8.

S17. Li, H.; Lv, C.-T.; Li, Y.-T.; Gao, G.-Y.; Meng, Y.-F.; You, Y.-L.; Tian, Q.; Liang, K.-Q.; Chen, Y.; Chen, H.; et al. RNA-sequencing transcriptome analysis of Avicennia marina (Forsk.) Vierh. leaf epidermis defines tissue-specific transcriptional response to salinity treatment. Sci. Rep. 2023, 13, 7614. https://doi.org/10.1038/s41598-023-34095-x.

S18. Alabbasi, G.B. Effect of foliar application with ferrous sulphate and AG3 on saplings growth of sour orange (Citrus aurantium L.). Plant Arch. 2019, 19, 4215–4218.

S19. Ren, R.; Wang, M.; Zhang, L.; Ren, F.; Yang, B.; Chen, H.; Zhang, Z.; Zeng, Q. Manganese biofortification in grapevine by foliar spraying improves volatile profiles of Cabernet Sauvignon grapes and wine sensory traits. Food Chem. X 2025, 25, 102150. https://doi.org/10.1016/j.fochx.2024.102150.
